# Golgi-localized membrane protein AtTMN1/EMP12 functions in the deposition of rhamnogalacturonan II and I for cell growth in Arabidopsis

**DOI:** 10.1093/jxb/erab065

**Published:** 2021-02-15

**Authors:** Akihiko Hiroguchi, Shingo Sakamoto, Nobutaka Mitsuda, Kyoko Miwa

**Affiliations:** 1 Graduate School of Environmental Science, Hokkaido University, Sapporo 060-0810, Japan; 2 Bioproduction Research Institute, National Institute of Advanced Industrial Science and Technology (AIST), Tsukuba 305–8566, Japan; 5 Forschungszentrum Jülich, Germany

**Keywords:** *Arabidopsis thaliana*, boron, cell elongation, cellular attachment, endomembrane protein (EMP), Golgi apparatus, pectin deposition, rhamnogalacturonan I, rhamnogalacturonan II, transmembrane nine protein (TMN)

## Abstract

Appropriate pectin deposition in cell walls is important for cell growth in plants. Rhamnogalacturonan II (RG-II) is a portion of pectic polysaccharides; its borate crosslinking is essential for maintenance of pectic networks. However, the overall process of RG-II synthesis is not fully understood. To identify a novel factor for RG-II deposition or dimerization in cell walls, we screened Arabidopsis mutants with altered boron (B)-dependent growth. The mutants exhibited alleviated disorders of primary root and stem elongation, and fertility under low B, but reduced primary root lengths under sufficient B conditions. Altered primary root elongation was associated with cell elongation changes caused by loss of function in *AtTMN1* (*Transmembrane Nine 1*)/*EMP12*, which encodes a Golgi-localized membrane protein of unknown function that is conserved among eukaryotes. Mutant leaf and root dry weights were lower than those of wild-type plants, regardless of B conditions. In cell walls, *AtTMN1* mutations reduced concentrations of B, RG-II specific 2-keto-3-deoxy monosaccharides, and rhamnose largely derived from rhamnogalacturonan I (RG-I), suggesting reduced RG-II and RG-I. Together, our findings demonstrate that AtTMN1 is required for the deposition of RG-II and RG-I for cell growth and suggest that pectin modulates plant growth under low B conditions.

## Teaser

Arabidopsis TMN1, a Golgi-localized membrane protein conserved among eukaryotes, is required for pectic polysaccharide deposition in cell walls for cell growth.

## Introduction

In multicellular organisms, tissue and organ formation is mediated by cellular adhesion. In plants, the stiffness and plasticity of cell walls support cellular growth and organization. Pectin, which is found within primary cell walls as a gel matrix, is critical for cell adherence (Daher and Braybrook, 2015).

Pectin consists of galacturonic acid (GalA)-rich polysaccharides, including homogalacturonan (HG), rhamnogalacturonan-I (RG-I), and rhamnogalacturonan-II (RG-II) domains (Voragen *et al.*, 2009). HG is a major pectin component composed of a polymer of (α-1,4)-linked d-galacturonic acids. De-methylesterified HG chains are crosslinked through ionic bonding between calcium ion (Ca^2+^) and carboxyl residues. This crosslinking of HG and Ca^2+^ is important for normal pectin network maintenance, which controls pectin gel properties. RG-I represents the second largest fraction of pectin; it contains a backbone consisting of repeating (α-1,2)-l-rhamnose-(α-1,4)-d-galacturonic acid and side chains. RG-II is a minor but highly conserved component in plants; it consists of a HG backbone linked to four distinct side chains composed of more than 12 different monosaccharides (O’Neill *et al.*, 2004). RG-II is crosslinked with borate between the two RG-II monomers, to form borate-dimerized RG-II through the formation of *cis*-diol ester bonds with apiosyl residues in side chain A. Boron (B) depletion has been reported to cause cellular adhesion and cell elongation disorders in plants through a reduction in the quantity of crosslinked RG-II (Ishii *et al.*, 2001; Iwai *et al.*, 2002; Chatterjee *et al.*, 2014; Camacho-Cristobal *et al.*, 2015).

Several proteins involved in pectin biosynthesis have been characterized. RG-II structure is synthesized in the Golgi apparatus by a variety of glycosyltransferases, using nucleotide diphosphate-linked sugars as activated donor substrates (Driouich *et al.*, 2012). Most nucleotide diphosphate-linked sugars are synthesized in the cytosol (Ahn *et al.*, 2006; Reboul *et al.*, 2011) and then transported into the Golgi (Sechet *et al.*, 2018). However, thus far, the overall molecular mechanism underlying RG-II polysaccharide synthesis in the Golgi and subsequent secretion to the apoplast remains poorly understood.

Recent studies have shown that the transmembrane nine (TMN) protein family is involved in cellular attachment among eukaryotes including *Drosophila melanogaster*, *Dictyostelium discoideum*, *Saccharomyces cerevisiae*, and *Homo sapiens* (Cornillon *et al.*, 2000; Bergeret *et al.*, 2008; Froquet *et al.*, 2008; Paolillo *et al.*, 2015). TMNs belong to a superfamily of nine multi-spanning endomembrane proteins; they are highly evolutionarily conserved (Hegelund *et al.*, 2010). TMN proteins contain a long N-terminal domain with variable amino acid sequences, followed by nine transmembrane domains and a short tail on the C-terminus. TMN proteins in *Dictyostelium* and *Drosophila* interact with integrin-like protein and peptidoglycan recognition protein, respectively, for transport into the plasma membrane (Froquet *et al.*, 2012; Perrin *et al.*, 2015a, b). TMN proteins are localized to the Golgi apparatus in *Dictyostelium* and to intracellular vesicles or plasma membrane in *Drosophila*. TMN proteins may be involved in vesicular trafficking systems; they commonly contribute to cellular attachment among eukaryotes, despite their distinct interacting proteins and sub-cellular localization in different organisms. The presence of TMN is also conserved in plants; the Arabidopsis and *Oryza sativa* genomes have 12 and 17 paralogs, respectively. AtTMN1/EMP12 is localized to the Golgi apparatus (Gao *et al.*, 2012); however, its physiological function in plants has not yet been revealed.

In this study we applied forward genetics to identify a new component required for RG-II deposition or dimerization in cell walls, focusing on the effects of altering B-dependent growth patterns, especially under low B conditions. This is because some RG-II synthesis mutants have shown impaired growth in response to changes in B concentrations caused by changes in the efficiency of borate-dimerized RG-II formation (O’Neill *et al.*, 2001; Voxeur *et al.*, 2011; Sechet *et al.*, 2018). Through characterization of the isolated mutants, we revealed that loss of function of *AtTMN1* partially relieved inhibition in root elongation under severely low B, and constantly reduced overall biomass compared with wild type plants, regardless of B conditions. It was suggested that the amounts of RG-II and RG-I were reduced in the cell wall of *tmn1* mutants, highlighting a role of Arabidopsis TMN1 in deposition of pectin in cell walls, for normal plant growth.

## Materials and Methods

### Plant materials and growth conditions

*Arabidopsis thaliana* (L.) Heynh. accession Columbia-0 (Col-0) was used as the wild type (WT). Plants were grown in solid or liquid media (Fujiwara *et al.*, 1992). Solid media contained 1% (w/v) sucrose and 1% (w/v) gellan gum (FUJIFILM Wako Pure Chemical, Japan). Boron (B) concentrations were adjusted with boric acid. For growth in solid media, surface-sterilized seeds were incubated in ultrapure water at 4 °C for stratification. Seeds were sown on solid media containing 0.1 μM (severely low B) or 100 μM (normal B) boric acid. They were incubated in a vertical position at 22 °C under a 16 h light/8 h dark cycle; light was provided by white fluorescent lamps (7 000–8 000 lux). To analyse vegetative growth, total B concentration in plant tissues and cell wall properties, Arabidopsis plants were germinated on rockwool with ultrapure water, and then incubated for 7 d. Seedlings were transferred into liquid media containing 0.1 µM (severely low B), 0.3 µM (mildly low B), 100 µM (normal B), and 500 μM (high B) boric acid; they were incubated for 38 d for vegetative growth and total B concentration analysis, and for 37 d for cell wall analysis. During the initial 14 d after the transfer, liquid medium was replaced weekly; subsequently, they were replaced at intervals of 3 d. Plants were grown at 70% relative humidity at 22 °C under short days (10 h light/14 h dark) to continue the vegetative growth stage. For reproductive growth tests, after plants had been incubated in water on rockwool for 7 d, they were grown for 68 d in liquid media supplemented with 0.1 µM, 0.3 µM, 100 µM, and 500 μM boric acid under long days (16 h light/8 h dark). Liquid medium was replaced weekly during the initial 14 d after transfer, and subsequently at intervals of 3 d or 4 d.

### Mutant screening and genotyping of *tmn1-1, tmn1-2*, and *tmn1-3* mutants

For mutant screening, Col-0 seeds were mutagenized with ethyl methanesulfonate; 12 400 mutagenized M_2_ seeds were grown on solid media containing 0.03 μM boric acid, representing severe B deficiency and 114 plants showing longer roots were initially selected. Then, M_3_ seeds derived from self-pollination of the selected M_2_ plants, were grown on solid media under 0.03 μM and 100 μM B and mutant plants were screened for those showing longer roots compared with Col-0 under 0.03 μM (low B), but did not exhibit longer roots under 100 µM boric acid (i.e., sufficient B) supply. Among 22 mutant candidates, two mutant lines, numbers 19 (*tmn1-1)* and 45 (*tmn1-2*), were investigated in this study. Isolated mutants 19 and 45 were crossed with Landsberg *erecta* (L*er*) and genetic mapping was conducted. The whole genome of each mutant was re-sequenced using the HiSeq2000 platform (Illumina, USA). The data were analysed using the DNA Data Bank of Japan Read Annotation Pipeline (Nagasaki *et al.*, 2013) and the candidates responsible for mutations were found.

Single-nucleotide polymorphisms in *AtTMN1* were detected by dCAPS and CAPS markers for *tmn1-1* and *tmn1-2*, respectively. DNA fragments of the *AtTMN1* sequence were amplified by PCR using Primer 1 (P1) and P2 for *tmn1-1*, and P3 and P4 for *tmn1-2* (Supplementary Table S5). The resulting fragments were then digested with *Taq*I for *tmn1-1* and *Mbo*I for *tmn1-2*.

*tmn1-3* (WiscDsLoxHS217_03C), a T-DNA insertion line of *AtTMN1*, was originated from Col; the seeds were obtained from the Arabidopsis Biological Resource Center (USA). From the segregating population, plants homozygous for T-DNA were established by PCR using P5 and P6 for T-DNA insertions, and P6 and P7 for the WT genome (Supplementary Table S5). 

### Detection of the full-length transcript of *AtTMN1*

Full-length transcripts of *AtTMN1* were detected by reverse transcription PCR. Plants were grown in solid media containing 100 μM boric acid for 11 d. Total RNA was extracted from leaves and roots using the RNeasy Plant Mini Kit (Qiagen, Germany). cDNA was synthesized using Prime Script RT Enzyme Mix (Takara, Japan). cDNA of *AtTMN1* and *Actin1* (cDNA quality control) were amplified using KOD-Plus-Neo (Toyobo, Japan) with specific primers P8 and P9 for *AtTMN1,* and P10 and P11 for *Actin1* (Supplementary Table S5). cDNA from 5 ng (for *AtTMN1* and *Actin1* in rosette leaves), 10 ng (for *AtTMN1* in roots), and 1 ng (for *Actin1* in roots) of total RNA was used as template; 40 cycles of amplification were performed.

### Measurement of cell lengths and cell numbers in roots

To obtain fully elongated cortical cell length measurements and meristematic cell counts, the root tips of plants grown on solid media were stained and observed. Under severely low B (0.1 μM), primary roots (PR) of 4-d-old plants were used, in which Col-0 had shorter roots than the *tmn1* mutants, but in which root tip cells had not severely collapsed. PRs were collected directly from solid media and stained with 10 μg ml^–1^ propidium iodide (PI; FUJIFILM Wako Pure Chemical, Japan) for 15 min, then rinsed twice in ultrapure water. Under normal B (100 μM), 11-d-old plants were used, in which root growth inhibition was obvious in the mutants. PRs were collected directly from solid media and stained with PI as described above for 40 min. Images were obtained using a confocal laser scanning microscope (LSM510, Zeiss, Germany). The excitation and detection wavelength windows were set at 488 and >540 nm, respectively. Up to three fully elongated cortical cells were selected from each PR; their longitudinal lengths were measured using ImageJ software (https://imagej.nih.gov/ij/). The numbers of cortical cells between the quiescent centre and the first elongating cortical cell were counted in the PI-stained PRs. Counting was performed using the Cell Counter ImageJ plugin (https://imagej.nih.gov/ij/plugins/cell-counter.html).

### Observation of columella cells in root tips

To examine root morphology, PR tips were stained as described above, with a slight modification to avoid root cap detachment during sampling. Plants were grown on solid media containing 100 μM boric acid for 14 d. PRs were cut; several drops of ultrapure water were placed on PRs while they remained on the media to ensure columella cells remained intact. Floating PRs were stained with PI for 5 min and observed by confocal laser scanning microscopy (LSM510, Zeiss, Germany).

### Plasmid construction and plant transformation

In preparation for the complementation test and observation of the sub-cellular localization of GFP-AtTMN1 protein, we generated new transgenic lines carrying *proAtTMN1:SP(AtTMN1)-GFP-AtTMN1genome* referring to a design described by Gao *et al.*, (2012). This construct was to express *GFP-AtTMN1* under the control of the endogenous *AtTMN1* promoter. Genomic nucleotides 3657117–3664330 of chromosome 1 sequences consisting of the putative promoter region (2.1 kb), ORF (4.3 kb), and terminator (0.7 kb), were amplified using Prime STAR HS DNA Polymerase (Takara, Japan) with primers P12 and P13 (Supplementary Table S5). The fragments were cloned into the pENTR/D-TOPO vector using Gateway technology (Invitrogen, USA), and the plasmid was named pAH2. To construct *proAtTMN1:SP(AtTMN1)-GFP-AtTMN1genome*, three PCR fragments were assembled. The first fragment, which included a sequence encoding the AtTMN1 signal peptide (SP), was amplified from pAH2 using PCR, with primers P14 and P15 (Supplementary Table S5). The second fragment, which included *GFP*, was amplified using PCR, with primers P16 and P17 (Supplementary Table S5). The third fragment, which included *AtTMN1* coding sequences, was amplified from pAH2 using PCR, with primers P18 and P19 (Supplementary Table S5). The three fragments were mixed and then amplified using primers P14 and P19 (Supplementary Table S5). The sequences encoding GGGGS between SP (26 aa) and GFP, and GGGSGGGS between GFP and AtTMN1 (27 aa) were inserted as linkers using the underlined primer sequences shown in Supplementary Table S5. The resulting fragments were digested with *Sca*I and *Bam*HI; they were then ligated into the pAH2 vector, which had been treated with both enzymes. The resulting plasmid, *proAtTMN1:SP(AtTMN1)-GFP-AtTMN1genome* in pENTR/D-TOPO, was named pAH3. The insert in pAH3 was cloned into the destination vector pMDC99 (Curtis and Grossniklaus, 2003) by means of an LR reaction using Gateway technology (Invitrogen, USA), and the plasmid was named pAH6. pAH6 was introduced into *Agrobacterium tumefaciens* strain GV3101 (C58C1Rif^r^) pMP90 (Gm^r^), and *tmn1-1* plants were transformed using the floral dipping method (Clough and Bent, 1998). *proAtTMN1:SP(AtTMN1)-GFP-AtTMN1genome* was confirmed to be functional by testing the restoration of root growth inhibition of *tmn1-1* in the T_2_ generation under 0.1 μM B. Among 30 independent transgenic lines, 20 exhibited full or partial complementation. For detailed analysis, a T_3_ generation homozygous for T-DNA was established and four independent lines were used: pAH6-90-2, pAH6-100-1 (#1), pAH6-102-3, and pAH6-151-2 (#2).

### Preparation of cell wall fractions from Arabidopsis rosette leaves and roots

For cell wall property analysis, alcohol-insoluble residues (AIRs) were prepared as previously described (Matsunaga and Ishii, 2006). Rosette leaves and roots were harvested from 44-d-old plants and then frozen. For each analysis, the following number of plants were collected as a single sample: B concentration, six for rosette leaves and four to nine for roots; ratio of dRG-II-B to total RG-II, six each for rosette leaves and roots; 2-keto-3-deoxy monosaccharide concentration, three to six for rosette leaves and six to 12 for roots; monosaccharide composition, six for rosette leaves. The frozen materials were homogenized in 80% (v/v) ethanol. After the homogenates had been centrifuged, all insoluble pellets were washed with 80% (v/v) ethanol twice, 99.5% (v/v) ethanol once, chloroform/methanol (1:1, v/v) twice, acetone once, and ultrapure water twice. The AIRs were freeze-dried with FDU-1200 (EYELA, Japan) and treated as cell wall fractions. For monosaccharide composition analysis, rosette leaves were homogenized in 80% (v/v) ethanol. After the homogenates had been centrifuged, insoluble debris were washed with 80% (v/v) ethanol twice, 80% (v/v) ethanol overnight, 99.5% (v/v) ethanol once, chloroform/methanol (1:1, v/v) three times, acetone twice, and ultrapure water twice. The AIRs were freeze-dried as described above.

### B and Ca concentration measurements

To measure total B concentrations, rosette leaves and roots of 45-d-old plants were harvested and rinsed with ultrapure water. The harvested rosette leaves and roots were dried at 60 °C for longer than 2 d and the dry weights were measured. After the samples had been dried, they were submerged in concentrated HNO_3_ (60–61%) at 22–24 °C for 2 d, digested at 110 °C, then completely digested with H_2_O_2_ (30–35.5%) at 80 °C. The digested samples were dissolved in 2% HNO_3_. B concentrations were measured by inductively coupled plasma mass spectrometry (ELAN 6100 DRC-e, PerkinElmer, USA). To measure B and Ca concentrations in cell walls in rosette leaves and roots, 0.7–2.3 mg of AIR samples were submerged in concentrated HNO_3_ at 22–24 °C for 2–4 d. The samples were digested as described above and then dissolved in 2% HNO_3_. B and Ca concentrations were then determined by inductively coupled plasma mass spectrometry.

### Determination of RG-II-B dimer formation in cell walls

To determine the ratio of dRG-II-B (RG-II-B dimer) to total RG-II in rosette leaf and root cell walls, the RG-II dimers and monomers were analysed as previously described (Matsunaga and Ishii, 2006) with slight modifications; 2.0–2.2 mg and 1.0–2.1 mg of AIRs were used for rosette leaves and roots, respectively. AIRs were saponified with 300 μl of 0.1 M NaOH at 4 °C for 4 h to remove methyl and acetyl esters; the supernatant pH was then adjusted to 5.0 with 10% (v/v) acetic acid. The suspensions were treated with six units (U) of *endo*-polygalacturonase (EPG) M1 for 24 h at 4 °C to release RG-II from AIRs. Before use, EPG from *Aspergillus niger* (Megazyme, Ireland) was dialysed with 0.1 M sodium acetate buffer. After the suspensions had been centrifuged, the supernatants were filtered through 0.45 μm membranes and subjected to size-exclusion HPLC/refractive index detection (HITACHI High Technologies Corporation, Japan) using a Diol-120 column (8 mm × 300 mm, YMC Co., Kyoto, Japan). The analyses were performed as follows: eluent, 0.2 M ammonium formate (pH 6.5); flow rate, 1.0 ml min^–1^; and injection volume, 10 μl or 100 μl for rosette leaves and 100 µl, 150 µl, or 200 μl for roots. Relative proportions of dRG-II-B to total RG-II were calculated from the peak area of dRG-II-B and RG-II monomer.

### Determination of 2-keto-3-deoxy sugars in cell walls

To estimate the quantities of RG-II in rosette leaf and root cell walls, 2-keto-3-deoxy (RG-II specific) sugars were measured using a modified thiobarbituric acid method (York *et al.*, 1985). Approximately 2.0 mg and 1.5 mg of AIRs were used for rosette leaves and roots, respectively. AIRs were saponified with 190.5 μl of 0.1 M NaOH at 4 °C for 8 h; the supernatant pH was adjusted to 5.5 with 10% (v/v) acetic acid. Cell walls were treated with 5 U of EPG M2 from *Aspergillus aculeatus* (Megazyme, Ireland) for 89 h at 35 °C for complete digestion. Before use, the enzyme was dialysed with 0.1 M sodium acetate buffer. The suspensions were centrifuged and the supernatants were collected. This process was repeated three times to completely remove insoluble residues. The supernatant (200 μl) was mixed with 100 μl of 0.5 M H_2_SO_4_, then incubated for 30 min at 100 °C to hydrolyse polysaccharides into monosaccharides. The solutions were cooled for 10 min at 22–24 °C. Following this, 250 µl of 40 mM HIO_4_ dissolved in 62.5 mM H_2_SO_4_, was added to the solution, which was then incubated for 20 min at 22–24 °C to generate formylpyruvic acid from oxidized 2-keto-3-deoxy sugars. Subsequently, 500 µl of 2% Na_2_SO_3_ dissolved in 0.5 M HCl, was added to the solutions to neutralize excess HIO_4_. Then 500 µl of 25 mM thiobarbituric acid was added to the solutions, which were then incubated for 15 min at 100 °C to generate pigments. DMSO (99.5%, 1 ml) was added to the solutions, which were then incubated for 6–7 min at 22–24 °C to stabilize the pigments. The 548 nm absorbance of the pigments was measured using a spectrophotometer (U-3900/3900H, HITACHI High Technologies Corp., Japan). The quantities of 2-keto-3-deoxy sugars were calculated using 2-keto-3-deoxyoctonate ammonium salt (Sigma-Aldrich, USA) as a standard.

### Observation of pectin distribution in cross-sections of roots

To observe pectin distribution, the PRs were embedded in LR White resin (Nisshin EM Corp., Japan); pectin was visualized with an indirect immunofluorescence method. Plants were grown on solid media containing 0.1 µM and 100 μM boric acid for 5 d. The PR tips were cut and then immersed with 4% paraformaldehyde dissolved in 20 mM sodium cacodylate buffer (pH 7.4) for 2 h at 4 ºC to fix cellular structures. The roots were immersed with 50% ethanol for 30 min at 4 ºC to be dehydrated. The process was performed with 60, 70, 80, and 90% ethanol for 30 min at each step, followed by 95% ethanol for 21 h at 4 ºC. Finally, the roots were immersed with absolute ethanol for 1 h at 4 ºC. Then the roots were infiltrated with 50% and 100% LR White resin for 22.5 and 47 h, respectively, at 4 ºC. After transferring the roots into 100% LR White resin in 1.5 ml plastic tubes, the resins were incubated for 32 h at 60 ºC to polymerize completely. Serial root sections (0.99 μm) were prepared using an ultramicrotome (ULTRACUT N, Reichert-Nissei, Germany) and placed on coated glass slides (MAS-01, Matsunami Glass Industry, Japan ). Section samples were treated with 2 N HCl for 15 min at 22–24 °C for epitope activation and then washed three times with phosphate-buffered saline (PBS: 137 mM NaCl, 2.7 mM KCl, 10 mM Na_2_HPO_4_, 2 mM KH_2_PO_4_, pH 7.4; FUJIFILM Wako Pure Chemical, Japan) for 42–6 (anti-RG-II antibody; Zhou *et al.*, 2018) or T/Ca/S buffer (20 mM Tris-HCl, pH 8.2, 0.5 mM CaCl_2_, 150 mM NaCl) for 2F4 (anti-Ca^2+^ cross-linked HG antibody; PlantProbes, UK). For blocking, the sections were incubated with 10% normal goat serum (Thermo Fisher Scientific, USA) for 1 h at 22–24 °C and were then labelled with primary antibodies: 20 μg ml^–1^ anti-RG-II antibody, 42–6, and 1/10 anti-Ca^2+^ cross-linked HG antibody, 2F4 dissolved in 10% normal goat serum. They were incubated for 20 h at 4 ºC. After washing with the buffers three times, the sections were incubated with 20 μg ml^–1^ (1/100) goat anti-rabbit IgG (H+L) and 20 μg ml^–1^ (1/100) goat anti-mouse IgG1 antibodies conjugated to Alexa Fluor 488 (Thermo Fisher Scientific, USA) dissolved in 10% normal goat serum against 42–6 and 2F4, respectively, for 2 h at 22–24 °C. Following washing with the buffers three times, the sections were mounted with 50% (v/v) glycerol to be observed with a fluorescence microscope (DM2500, Leica, Germany). The Alexa Fluor 488 was excited with a mercury fluorescence source (EBQ 100-04-L, Leistungselektronik JENA GmbH, Germany) and observed through a 515 nm long-pass emission filter.

### Cell wall monosaccharide composition analysis

To determine the monosaccharide composition of cell walls, extracted rosette leaf cell walls were analysed using an ultra-performance liquid chromatography–*p*-aminobenzyl ethyl ester system (Sakamoto *et al.*, 2015). Approximately 2.00–3.00 mg of AIR were added to a 2 ml microtube, and starch contained in the AIR was digested with 1 ml of an amylase solution containing 500 U ml^–1^ of α-amylase from porcine pancreas (Megazyme, Ireland) and 0.33 U ml^–1^ of amyloglucosidase from *Aspergillus niger* (Megazyme, Ireland) in 0.1 M sodium malate buffer (pH 6.0) at 37 °C for 18 h. Insoluble residues in the amylase suspension were rinsed with absolute ethanol and ultrapure water, then dried completely at 60 °C overnight. The dried pellet was depolymerized with 50 μl of 72% (w/w) H_2_SO_4_ for 1 h with shaking at 1700 rpm. After the addition of 1.4 ml of ultrapure water to the AIR suspension, the AIR was hydrolysed at 121 °C for 1 h. The hydrolysed supernatant was neutralized with calcium carbonate powder and adjusted to around pH 5.0. Monomerized sugars in the neutralized supernatant were labelled with aminobenzyl ethyl ester solution containing 330 mg ml^–1^ of *p*-aminobenzyl ethyl ester (FUJIFILM Wako Pure Chemical, Japan), 66 mg ml^–1^ of sodium cyanoborohydride, 8% (v/v) acetic acid, and 75% (v/v) methanol at 80 °C for 30 min. Chromatographic separation and detection were conducted using an ACQUITY UPLC H-Class system equipped with an ACQUITY UPLC BEH C18 column (100 mm × 2.0 mm, 1.7 μm particle size, Waters Corp., USA) and fluorescence detector (ACQUITY UPLC FLR Detector, Waters Corp., USA) as previously described (Sakamoto *et al.*, 2015). For samples without amylase treatment, AIR powder was hydrolysed with H_2_SO_4_; monosaccharide composition in hydrolysate was analysed as described above.

### Observation of GFP-AtTMN1 localization

To examine the molecular function of AtTMN1 protein, GFP-AtTMN1 localization was observed in the presence of exocytosis and endocytosis inhibitors using a confocal laser scanning microscope (TCS SP5, Leica, Germany). Transgenic plants carrying *proAtTMN1:SP(AtTMN1)-GFP-AtTMN1genome* were grown for 5 d on solid media supplemented with 100 μM boric acid. In each treatment, chemicals were dissolved in liquid media containing 100 μM boric acid. Roots were incubated with 2 μM FM4-64 (Thermo Fisher Scientific, USA) for 5 min and then with 50 μM cycloheximide (Sigma-Aldrich, USA; a protein synthesis inhibitor) for 30 min. Subsequently, roots were treated with 50 μM brefeldin A (BFA; Sigma-Aldrich, USA; an exocytosis inhibitor) and cycloheximide for 60 min. The liquid media for BFA treatment contained 1.2% DMSO. To examine the possibility of AtTMN1 localization in the plasma membrane, 5-d-old transgenic plants grown under 100 μM B were treated with 100 μM Dynasore (Sigma-Aldrich, USA; an endocytosis inhibitor) for 90 min and then with 1 μM FM4-64 (Thermo Fisher Scientific, USA) for 30 s. The Dynasore liquid medium contained 1.7% DMSO. The laser excitation/detection wavelength bandwidths were 488/650–700 nm for FM4-64 and 488/500–530 nm for GFP.

To examine cell-type specific expression of AtTMN1 protein in roots, transgenic plants carrying *proAtTMN1:SP(AtTMN1)-GFP-AtTMN1genome* were grown for 5 d on solid media supplemented with 100 μM boric acid. PRs were collected directly from solid media and stained with 10 μg ml^–1^ PI for 1 min, then rinsed twice in ultrapure water. GFP and PI fluorescence in PRs was observed by the confocal laser scanning microscope. The laser excitation/detection wavelength bandwidths were 488/500–530 nm for GFP and 488/650–700 nm for PI. Their fluorescence was enhanced using Leica Application Suite Advanced Fluorescence Lite (2.6.0 build 7266; Leica, Germany).

### Co-expression analysis

Co-expression analysis was performed using publicly available transcriptome data including many tissues from various parts of plants (NCBI SRA Accession no.: PRJNA268115 [Klepikova *et al.*, 2015]; PRJNA314076 [Klepikova *et al.*, 2016]); leaf treated with abiotic stress (NCBI SRA Accession no.: PRJNA324514 [Klepikova *et al.*, 2016]), and many cell types of root (NCBI SRA Accession no.: PRJNA323955 [Li *et al.*, 2016]). Raw data was mapped to Arabidopsis TAIR10 CDS dataset by STAR software (Dobin *et al.*, 2013) and normalized globally by DESeq2 software (Love *et al.*, 2014) with default setting. Co-expressed genes showing Pearson’s correlation value *r*>0.6 with *AtTMN1* were collected (488 genes; Supplementary Table S3). Enrichment analysis was performed by binomial test of R statistical software.

### Statistical analyses

Statistical analyses were performed using R software ver. 4.0.2. PR lengths were compared among plant lines using the Tukey–Kramer test. Comparison between Col-0 and *tmn1* mutants in the other experiments were performed using Dunnett’s multiple comparison test.

## Results

### *AtTMN1* mutations altered primary root growth in response to B nutrition

**Figure 1.**
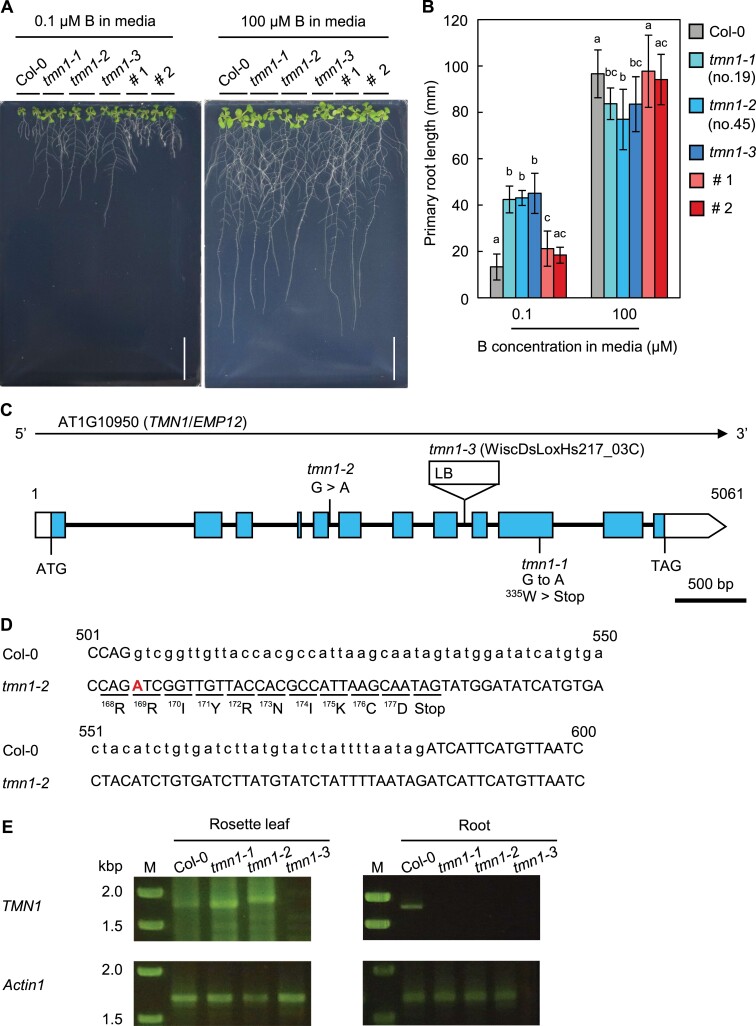
Impaired B nutrient response caused by loss of *AtTMN1* function. (A) Eleven DAS seedlings of Col-0, *tmn1-1* (no.19), *tmn1-2* (no.45), *tmn1-3*, and *tmn1-1* carrying *proAtTMN1:SP(AtTMN1)-GFP-AtTMN1genome* (lines #1 and #2) grown on solid media supplemented with 0.1 µM and 100 μM boric acid. Scale bars =20 mm. (B) Primary root lengths of seedlings 11 DAS under 0.1 µM and 100 μM B. Values are means ± SD of 16–20 individual plants. Different letters indicate significant differences among plant lines for each B condition (*P*<0.05, Tukey–Kramer test). (C) Gene structure of *AtTMN1* and mutation positions in *tmn1* alleles. Blue and white boxes indicate exons and untranslated regions, respectively. Black lines across exons indicate introns. Triangle indicates T-DNA insertion. Numbers correspond to the position of the genomic sequence from a transcriptional start site. (D) Insertion region of the fifth intron (81 bp) in *tmn1-2*. Uppercase and lowercase letters indicate exons and introns, respectively. A bold red letter represents a single-base substitution in *tmn1-2*. Numbers correspond to the position of the coding sequence from a translation start site. (E) *AtTMN1* transcripts (1.77 kb) amplified from cDNA in rosette leaves and roots using a *AtTMN1*-specific primer. *Actin1* full-length transcripts (1.69 kb) were amplified as a reference. M, DNA size marker.

To isolate novel components of RG-II deposition or dimerization, Arabidopsis Col-0 seeds were mutagenized with ethyl methanesulfonate; 22 mutant candidates with an altered response to B were isolated, which showed longer roots only under low B. Two independent mutants (nos.19 and 45) showed alleviation of root growth inhibition under low B, but exhibited reduced root growth under normal B conditions compared with WT Col-0 (Fig. 1A). The PR lengths of mutants nos.19 and 45 were 3.2-fold greater than the length of WT Col-0 under severely low (0.1 μM) B at 11 d after sowing (DAS; Fig. 1B). Under normal (100 μM) B, the PR lengths of mutants nos.19 and 45 were reduced by 13.4% and 20.4%, respectively, compared with Col-0; this indicated that B-dependent root growth was impaired in both mutants (Fig. 1B).

In the F_2_ population derived from F_1_ by crossing the mutants and WT L*er*, 19.7% and 14.8% of F_2_ plants exhibited the mutant phenotype (i.e. alleviation of inhibition of root elongation under low B) in mutants nos.19 and 45, respectively (Supplementary Table S1). Based on the segregation ratio, the phenotype appears to have been caused by a recessive mutation in a single genetic locus. Using a map-based cloning approach and genome analysis of the mutants, we found that both mutants carried a single-base substitution (G to A), but in a different position in *Transmembrane nine 1* (*AtTMN1/EMP12*; AT1G10950), which encodes a membrane protein of unknown function (Fig. 1C). AtTMN1 contains a long N-terminal domain followed by nine transmembrane domains, and is localized at the Golgi apparatus (Gao *et al.*, 2012). However, its mutant phenotypes have not been reported, and its physiological functions remain unknown.

Mutant no. 19 (*tmn1-1*) possesses a non-sense mutation in the 10th exon, which results in W355stop. Mutant no.45 (*tmn1-2*) carries a mutation in the 5’ splice donor site in the fifth intron (Fig. 1D). *tmn1-3*, a line carrying a T-DNA insertion in the eighth intron of *AtTMN1*, showed similar growth to *tmn1-1* and *tmn1-2* under 0.1 µM B and 100 μM B (Fig. 1A, B), indicating that *AtTMN1* mutations were responsible for the phenotype.

To examine *AtTMN1* mRNA in the *tmn1* mutants, full-length transcripts of *AtTMN1* were investigated using reverse transcription PCR. In rosette leaves, *AtTMN1* cDNA was detected in *tmn1-1* and *tmn1-2*, but not in *tmn1-3* (Fig. 1E). The detected PCR product in *tmn1-1* corresponded with the size of the *AtTMN1* PCR product in Col-0, whereas the size of the major product in *tmn1-2* was slightly larger. Sequence analysis showed that the fifth intron (81 bp), where the mutation was present at the 5’ splice donor site, was not spliced out; it remained within *tmn1-2* cDNA, resulting in a premature stop codon (Fig. 1D). Because proteins that are potentially translated from *tmn1-1* and *tmn1-2* mRNAs lack a large portion of the transmembrane domain at the C-terminus, functional AtTMN1 proteins are unlikely to be produced in *tmn1-1* and *tmn1-2* leaves. No transcript was detected in *tmn1* mutant roots (Fig. 1E), indicating that *AtTMN1* mRNA expression was below the detection limit in all three mutants, and that non-sense-mediated mRNA decay was induced in *tmn1-1* and *tmn1-2* in roots. Collectively, these results suggest that the *tmn1* mutants were loss-of-function mutants and that the mutant phenotypes were caused by loss of *AtTMN1* function.

To confirm that the loss of *AtTMN1* function was responsible for the mutant phenotypes, we conducted a complementation test by introducing a *proAtTMN1:SP(AtTMN1)-GFP-AtTMN1genome* construct into *tmn1-1* (no. 19), thereby expressing GFP-AtTMN1 fusion protein under the control of the native *AtTMN1* promoter. When T_3_ homozygous lines (#1 and #2) were grown under 0.1 and 100 μM B conditions, abnormal root growth in *tmn1-1* (no. 19) was rescued, further supporting the notion that impaired B-dependent growth was caused by the loss of *AtTMN1* function (Fig. 1A, B).

According to an Arabidopsis Electronic Fluorescent Pictograph Browser (Winter *et al.*, 2007), *AtTMN1* mRNA was universally detected in roots, rosette leaves, internodes and siliques during vegetative and reproductive growths. Publicly available transcriptome data suggests that *AtTMN1* is predominantly expressed in the mature xylem pole and cortex in roots (Supplementary Table S3). To examine cell-type expression of AtTMN1 in roots, GFP-AtTMN1 fluorescence was observed. GFP-AtTMN1 fluorescence was entirely detected in PR tips and weakly observed in the transition and mature zone where root hairs initiated. The fluorescence at the stele was relatively strong in any zone of the roots (Supplementary Fig. S1A, B, C, D). Furthermore, in PR tips, GFP-AtTMN1 was observed at the columella root cap; the intensity at a proximal end of root cap (Dolan *et al.*, 1993; Kumar and Iyer-Pascuzzi, 2020) tended to be higher compared with the other columella cells of distinct stages (Supplementary Fig. S1A, B). These results support that AtTMN1 is involved in root growth.

### Root cell elongation was altered in *tmn1* mutants

**Figure 2.**
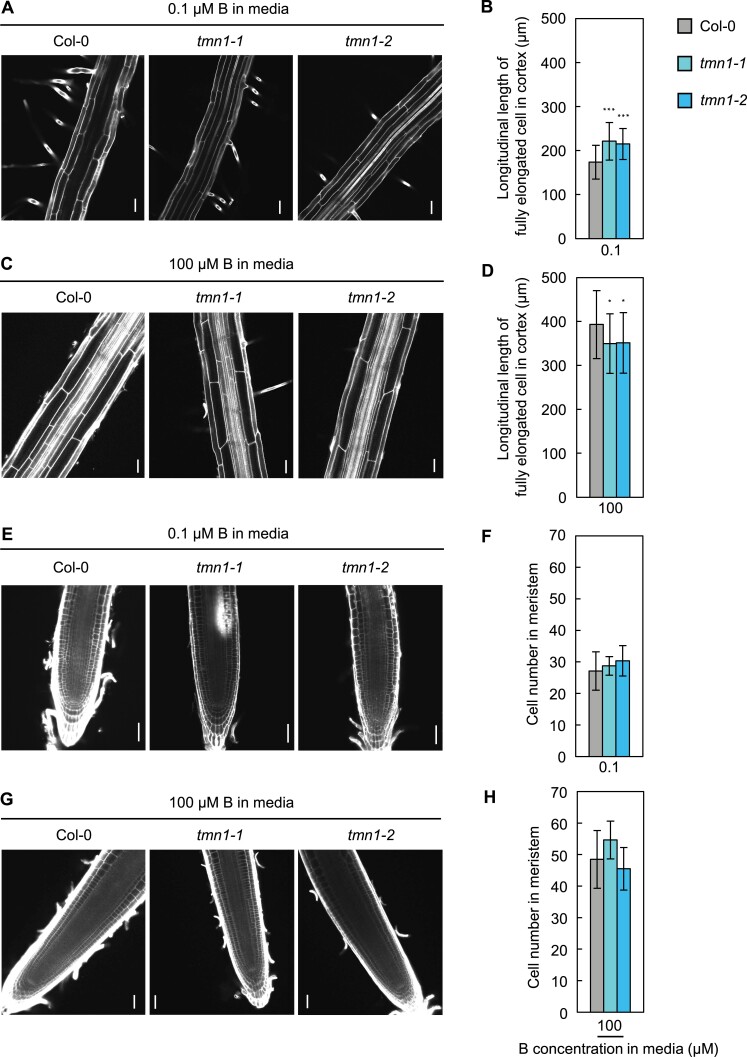
Increased and reduced cell elongation in the *tmn1* mutants under severely low and normal B conditions, respectively. (A, C) Representative confocal images of mature root cells stained with PI and (B, D) longitudinal lengths of fully elongated cortical cells of Col-0, *tmn1-1*, and *tmn1-2* at four DAS under 0.1 μM B (A, B) and at 11 DAS under 100 μM B (C, D). Values are means ± SD from 29–67 independent cells in 10–19 individual plants. (E, G) Representative confocal images of root meristematic cells stained with PI and (F, H) cortical cell numbers between the quiescent centre and the first elongating cortical cell in seedlings of Col-0, *tmn1-1*, and *tmn1-2* at four DAS under 0.1 μM B (E, F) and at 11 DAS under 100 μM B (G, H). Values are means ± SD from six to 15 individual plants. Scale bars =50 μm. Asterisks indicate significant differences between Col-0 and the *tmn1* mutants (*P<0.05, ***P<0.001; Dunnett’s multiple comparison test).

To understand which process was altered in *tmn1* mutant root growth, we measured the lengths of elongated cells in the root hair zone and counted cells in the meristematic zone at four DAS under 0.1 μM B and at 11 DAS under 100 μM B. Because incubation for longer than 4 d dramatically deformed root cell morphology under severely low B (0.1 μM), especially in Col-0, we observed the root cells at four DAS. Under 0.1 μM B, longitudinal lengths of fully elongated cortical cells were significantly increased by 31.2% and 27.3% in *tmn1-1* and *tmn1-2* (*P*<0.001; Fig. 2A, B), respectively, compared with Col-0. Conversely, under 100 μM B, cell lengths were significantly reduced by 11.0% and 10.6% in *tmn1-1* and *tmn1-2*, respectively (*P*<0.05; Fig. 2C, D). No significant differences in root meristem cell numbers were observed between Col-0 and the *tmn1* mutants under 0.1 µM or 100 μM B treatments (*P*>0.05; Fig. 2E–H). Our observations suggested that cell division was not primarily changed, but that cell elongation was affected in *tmn1* mutants under low and normal B conditions. Changes in cell length were positively correlated with changes in PR length in the *tmn1* mutants (Figs 1, 2); this suggested that the abnormal root growth observed in *tmn1* mutants was caused by changes in cell elongation.

The root tip morphology of seedlings grown in solid media under 100 μM B at 14 DAS showed that detaching root cap layers remained suspended from root tips in the *tmn1* mutants, but not in Col-0 (Supplementary Fig. S2). This finding suggested a defect in the process of root cap maturation in *tmn1* mutants, in addition to altered cell elongation.

### Leaf and root dry weights were reduced in *tmn1* mutants in hydroponic culture

**Figure 3.**
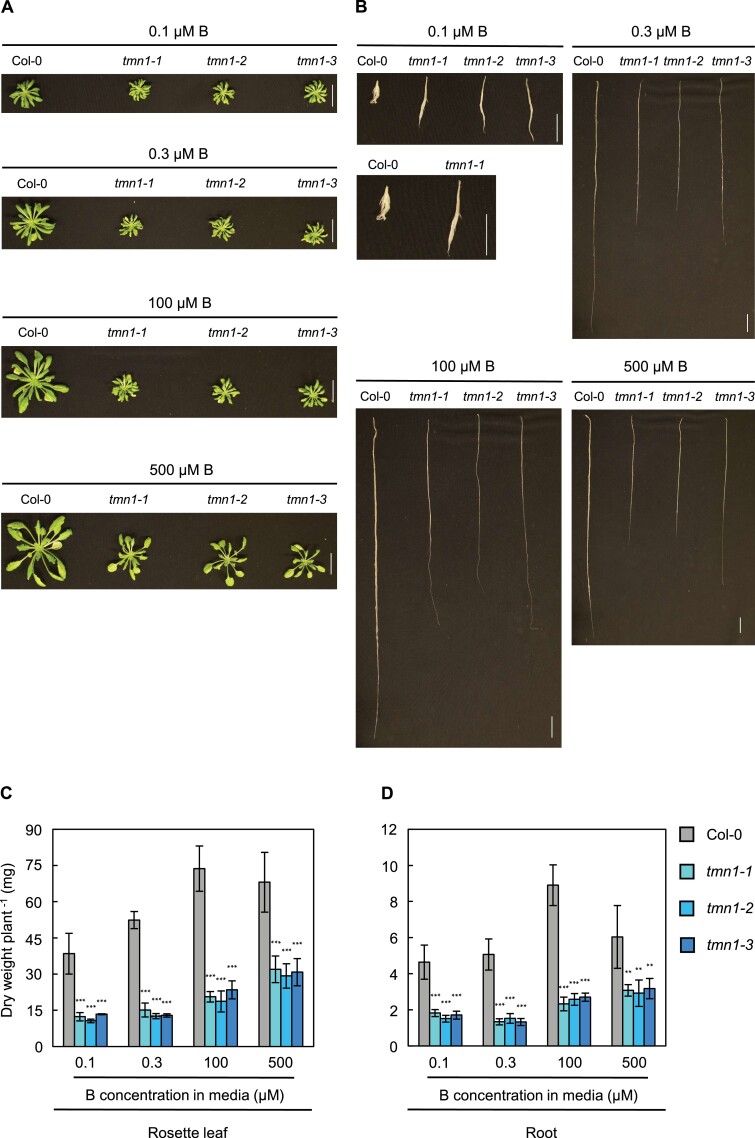
Defective vegetative growth in the *tmn1* mutants. Rosette leaves (A) and roots (B) of Col-0, *tmn1-1*, *tmn1-2*, and *tmn1-3* plants grown hydroponically for 44 d under 0.1 µM, 0.3 µM, 100 µM, and 500 μM B treatments under short days. Scale bars =20 mm. Dry weights of rosette leaves (C) and roots (D) in Col-0, *tmn1-1*, *tmn1-2*, and *tmn1-3* grown for 45 d. Values are means ± SD from four independent plants. Asterisks indicate significant differences between Col-0 and the *tmn1* mutants under each B treatment (**P<0.01, ***P<0.001; Dunnett’s multiple comparison test).

To investigate long-term vegetative growth in the *tmn1* mutants, plants were grown hydroponically under short days. Rosette leaf sizes were markedly reduced in *tmn1* mutants, compared with Col-0, under all B treatments (Fig. 3A). Roots were longer in the *tmn1* mutants than in Col-0 under severely low B (0.1 μM), whereas the roots of *tmn1* mutants were shorter than the roots of Col-0 under 0.3 µM (mildly low), 100 µM (normal), and 500 μM (high) B conditions (Fig. 3B); these findings were consistent with mutant root phenotypes in solid media (Fig. 1A, B). Root bundles of the *tmn1* mutants appeared thinner than those of Col-0 under all B treatments (Fig. 3B). Thickness of individual root tips in *tmn1* mutants seemed to be reduced compared with Col-0 under 0.1 µM and 100 μM B (Supplementary Fig. S3). Leaf and root dry weights were significantly reduced by 53.0–76.0% and 47.3–73.8% in the *tmn1* mutants under all B treatments including 0.1 μM B (*P*<0.01), when longer PRs were observed in the *tmn1* mutants than in Col-0 (Fig. 3C, D). This result indicated that overall biomass production in the vegetative stage was inhibited in the *tmn1* mutants, despite the presence of increased root elongation under severely low B treatment in the *tmn1* mutants.

To examine reproductive growth, plants were grown under long days. Although different growth responses to B deficiency by day length have been classically described (Warington, 1933), B-deficient symptoms in rosette leaf expansion and root elongation of Col-0 under low B did not appear to be largely affected by day length under our experimental regime. Similar to the vegetative growth results, the *tmn1* mutants displayed shorter roots under 0.3 µM (mildly low) B to 500 μM (high) B treatments, but longer roots under 0.1 μM (severely low) B treatment, compared with Col-0 (Supplementary Fig. S4C). Inhibited internode elongation and sterility were observed in Col-0 under severely low (0.1 µM) B and mildly low (0.3 μM) B treatments, whereas these disorders were partially recovered in the *tmn1* mutants, compared with Col-0 (Supplementary Fig. S4A, B), which suggested that B-deficiency symptoms were relieved in the *tmn1* mutants. However, main stem length and branch numbers were reduced in the *tmn1* mutants, compared with Col-0, under 100 µM (normal) B and 500 μM (high) B treatments (Supplementary Fig. S4A). Together, these results showed that AtTMN1 is necessary for normal growth in the vegetative and reproductive stages, irrespective of B conditions; moreover*, AtTMN1* mutations increase growth in specific tissues under low B conditions.

### Cell wall B concentrations were lower in rosette leaves and roots of the *tmn1* mutants

To explore the mechanisms of impaired growth in the *tmn1* mutants, total B concentration per dry weight was determined in plants hydroponically grown under short days. In rosette leaves, no significant differences in total B concentration were observed between Col-0 and the *tmn1* mutants under 0.1 µM, 0.3 µM, or 100 μM B treatments (*P*>0.05; Supplementary Fig. S5A). Under 500 μM B, leaf total B concentrations were significantly higher in *tmn1-1* compared with Col-0 (*P*<0.01), but not in *tmn1-2* or *tmn1-3* (*P*>0.05; Supplementary Fig. S5A). Because we observed no consistent changes in B concentrations in mutant leaves, changes in total B concentration among leaves were presumably not a primary cause of the biomass reduction observed in Fig. 3C. In roots, total B concentration significantly decreased in *tmn1-3* under 0.1 μM B, *tmn1-2* under 0.3 μM B, and all three *tmn1* mutants under 100 μM B, compared with Col-0 (*P*<0.05; Supplementary Fig. S5B). No significant differences were observed between Col-0 and the *tmn1* mutants under 500 μM B (*P*>0.05; Supplementary Fig. S5B). Reduced B concentrations in roots under normal B conditions may have caused the reduction in root growth observed in the *tmn1* mutants (Fig. 3D); however, reductions in root biomass observed under 500 μM B is not likely to be explained by changes in total B concentration in roots.

**Figure 4.**
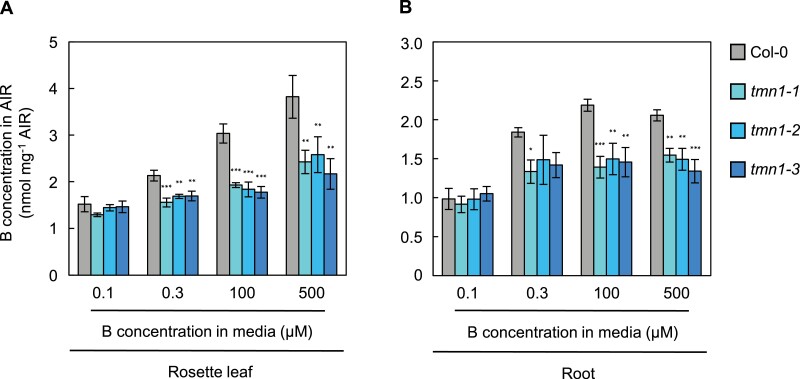
Reduced cell wall B concentrations in *tmn1* mutants under mildly low to high B conditions. B concentrations in rosette leaf (A) and root (B) cell walls of Col-0, *tmn1-1*, *tmn1-2*, and *tmn1-3* plants grown hydroponically under 0.1 µM, 0.3 µM, 100 µM, and 500 μM B treatments for 44 d under short days. For each cell wall sample, six individual plants for rosette leaves and four to nine plants for roots were harvested and homogenized. Values are means ± SD from three and four independent cell wall samples for rosette leaves and roots, respectively. Asterisks indicate significant differences between Col-0 and the *tmn1* mutants under each B condition (*P<0.05, **P<0.01, ***P<0.001; Dunnett’s multiple comparison test). AIR, alcohol-insoluble residue.

Because a primary physiological function of B in plants is pectic polysaccharide RG-II crosslinking in cell walls (O’Neill *et al.*, 2004), we next examined the AtTMN1 function in cell wall B concentration in plants hydroponically grown under short days. In rosette leaves, no significant differences in cell wall B concentration were observed between Col-0 and the *tmn1* mutants under 0.1 μM B (*P*>0.05; Fig. 4A). However, B concentrations were significantly (20.4–43.2%) lower in the *tmn1* mutants than in Col-0 in leaf cell walls under 0.3 µM, 100 µM, and 500 μM B treatments (*P*<0.01; Fig. 4A). In root cell walls, there were no significant differences between Col-0 and the *tmn1* mutants under 0.1 μM B (*P*>0.05; Fig. 4B). Under 0.3 μM B, B concentrations in root cell walls were significantly lower in *tmn1-1* (*P*<0.05); they also showed lower tendencies in *tmn1-2* and *tmn1-3* (Fig. 4B). Root cell wall B concentrations were significantly reduced by 24.9–36.3% in the *tmn1* mutants, compared with Col-0, under 100 µM and 500 μM B (*P*<0.01; Fig. 4B). Because B is predominately distributed to RG-II and nearly all RG-II is presumed to be crosslinked by borate in Col-0 under 100 µM B and 500 μM B treatments, the reduction of cell wall B concentration suggested that reduced quantities of borate-crosslinked RG-II were present in the *tmn1* mutants.

Calcium is another element in cell walls and it functions to cross-link de-methylesterified HG chains, a major domain of pectic polysaccharides. To verify the effects of AtTMN1 on Ca concentrations in cell walls, we analysed cell wall Ca concentrations. Rosette leaf cell wall Ca concentrations did not differ greatly between the *tmn1* mutants and Col-0 (Supplementary Fig. S6A). Although significant increments were observed in *tmn1-1* under 0.1 µM, 0.3 µM, and 500 μM B and in *tmn1-2* under 500 μM B (*P*<0.05; Supplementary Fig. S6A), these changes were not consistently observed in the leaf cell walls of *tmn1* mutants. In root cell walls, the *tmn1* mutants showed significant reductions in Ca concentration, compared with Col-0, under 0.1 µM and 0.3 μM B (*P*<0.05; Supplementary Fig. S6B). Under 100 µM and 500 μM B, there were no significant differences between Col-0 and the *tmn1* mutants (*P*>0.05; Supplementary Fig. S6B). In immunostaining with 2F4 which recognizes Ca^2+^ cross-linked HG, the signal at the lamella of cortex periphery tended to be decreased in 5-d-old *tmn1-1* and *tmn1-2* grown on solid media under 0.1 μM B compared with Col-0, suggesting potential reduction of Ca^2+^ cross-linked HG in the mutants under low B (Supplementary Fig. S6C).

Unlike the observed reduction in cell wall B concentrations, consistent patterns of changes in cell wall Ca concentrations were not found in the *tmn1* mutants, which suggested that the *AtTMN1* mutations predominately caused the observed reduction in cell wall B concentrations. Thus, reduced borate-cross-linked RG-II could be involved in the development of these impaired growth patterns.

### Cell wall RG-II was reduced in *tmn1* mutant rosette leaves and roots

We considered two explanations for the reduction of borate-dimerized RG-II based on lowered cell wall B concentrations in *tmn1* mutants (Fig. 4): (i) reduced efficiency of dimeric RG-II-B formation, and (ii) diminished total quantity of cell wall RG-II in the *tmn1* mutants. To test the first possibility, we determined the relative proportion of dRG-II-B to the proportion of total RG-II in cell walls of plants hydroponically grown under short days. In leaves, relative rates of RG-II cross-linking in cell walls were significantly higher in the *tmn1* mutants than in Col-0 under 0.1 μM B (*P*<0.05; Table 1). Under 0.3 µM, 100 µM, and 500 μM B, there were no significant differences in cross-linking rates between leaves of Col-0 and leaves of *tmn1* mutants (*P*>0.05; Table 1). In roots, cross-linking rates were significantly higher in *tmn1-3* under 0.1 μM B and in the three mutants under 0.3 μM B, compared with Col-0 (*P*<0.05; Table 1). Under 100 µM and 500 μM B, there were no significant differences between Col-0 and the *tmn1* mutants (*P*>0.05; Table 1). The lack of reduction in the relative proportions of dRG-II-B to total RG-II in the mutant cell wall likely refutes the hypothesis of lowered efficiency in dRG-II-B formation in the *tmn1* mutants.

**Table 1.** No reduction in the relative proportion of RG-II-B dimer formation in the *tmn1* mutants.

	Relative proportion of RG-II-B dimer in rosette leaf (%)				Relative proportion of RG-II-B dimer in root (%)			
Plant line	0.1 μM B in media	0.3 μM B in media	100 μM B in media	500 μM B in media	0.1 μM B in media	0.3 μM B in media	100 μM B in media	500 μM B in media
Col-0	78.0±3.5	85.9±1.5	91.1±5.5	93.8±1.7	62.1±4.2	72.2±3.2	92.5±0.4	90.4±4.1
*tmn1-1*	86.8±3.8^*^	84.6±3.7	87.8±2.0	91.3±2.2	71.3±5.8	81.7±1.9^**^	90.7±2.6	95.0±0.4
*tmn1-2*	85.1±1.8^*^	89.0±0.6	89.9±2.7	91.9±2.1	73.8±4.5	83.5±3.2^**^	92.9±3.3	93.3±3.7
*tmn1-3*	85.6±2.3^*^	89.5	91.9±2.2	91.9±3.6	75.3±6.2^*^	82.8±1.2^**^	90.9±2.1	92.7±0.3

Relative proportions of RG-II-B dimer in rosette leaf and root cell walls of Col-0, *tmn1-1*, *tmn1-2* and *tmn1-3*. The plants were grown hydroponically under 0.1 µM, 0.3 µM, 100 µM, and 500 μM B for 44 d under short days. For one individual cell wall sample, six plants for rosette leaves and roots were harvested to be homogenized. RG-II-B dimers and RG-II monomers released from AIRs by EPG were detected by size-exclusion HPLC/refractive index detector. Relative proportions represent percentages of RG-II-B dimer peak area which is divided by total area of RG-II-B dimers and RG-II monomers. Values represent means ± SD from three and four independent cell wall samples for rosette leaves and roots, respectively. The value of *tmn1-3* rosette leaf under 0.3 μM B represents a mean from two independent cell wall samples. Significant differences between Col-0 and the *tmn1* mutants under each B condition are indicated as *P<0.05, **P<0.01 (Dunnett’s multiple comparison test). AIR, alcohol-insoluble residue.

**Figure 5.**
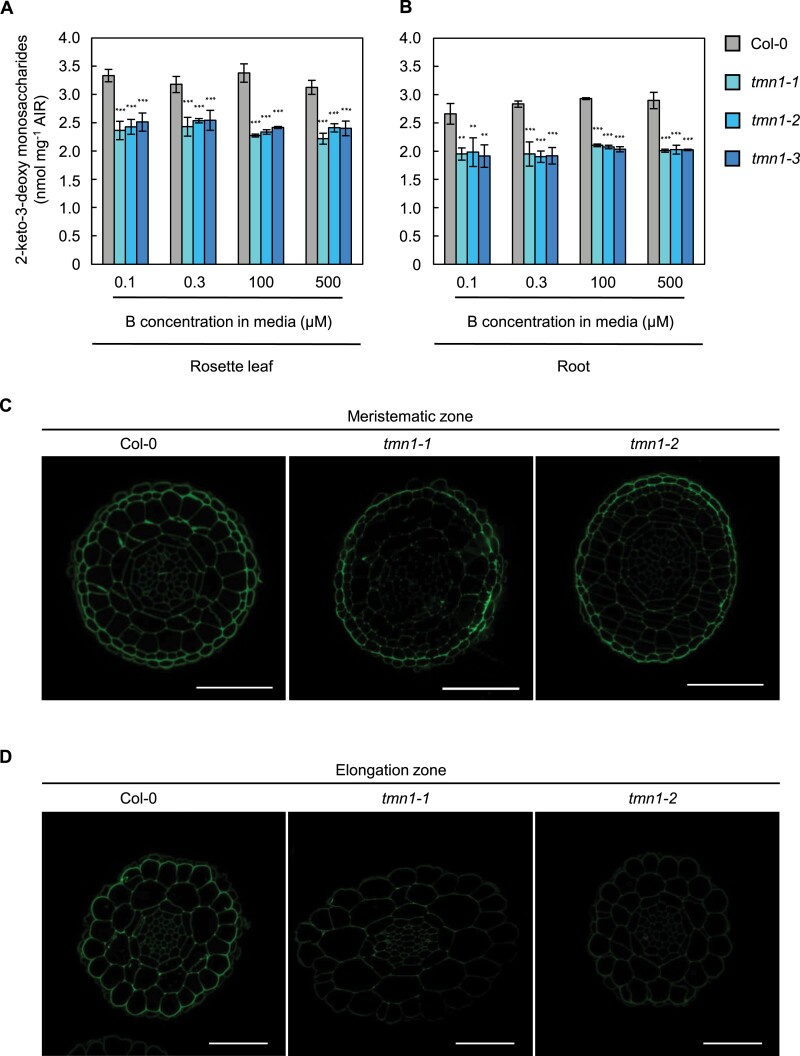
Reduced cell wall RG-II content in the *tmn1* mutants. Concentration of 2-keto-3-deoxy sugars in rosette leaf (A) and root (B) cell walls of Col-0, *tmn1-1*, *tmn1-2*, and *tmn1-3* plants grown hydroponically under 0.1 µM, 0.3 µM, 100 µM, and 500 μM B treatments for 44 d under short days. For one individual cell wall sample, rosette leaves of three to six plants and roots of six to 12 plants were harvested for homogenization. RG-II was solubilized from AIRs by EPG treatment and 2-keto-3-deoxy sugar concentrations were measured using a modified thiobarbituric acid method. Values are means ± SD from four independent cell wall samples. Asterisks indicate significant differences between Col-0 and the *tmn1* mutants under each B condition (**P<0.01, ***P<0.001; Dunnett’s multiple comparison test). The concentration of 2-keto-3-deoxy sugars was estimated based on the standard curve of 2-keto-3-deoxyoctonate ammonium salt. AIR, alcohol-insoluble residue. Immunohistochemistry of root cross-sections in the meristematic (C) and elongation zones (D) of PRs with an anti-RG-II antibody, 42–6. Plants were grown on solid media containing 100 μM boric acid for 5 d. Scale bars =50 μm.

To determine whether total quantities of RG-II were reduced in the *tmn1* mutants, 2-keto-3-deoxy sugars were measured in cell walls. In plants, 2-keto-3-deoxy sugars, (2-keto-3-deoxy-d-lyxo-heptulosaric acid and 3-deoxy-d-manno-oct-2-ulosonic acid) have been found only in RG-II; thus, their quantities represent the entire quantity of RG-II. The concentration of 2-keto-3-deoxy sugars in leaf cell walls were significantly reduced by 19.9–32.7% in the *tmn1* mutants, compared with Col-0, under all B treatments (*P*<0.001; Fig. 5A). Similarly, the concentration of 2-keto-3-deoxy sugars in root cell walls were significantly reduced by 25.5–32.8% in the *tmn1* mutants, compared with Col-0, under all B treatments (*P*<0.01; Fig. 5B). The reduced amount of 2-keto-3-deoxy sugars in cell walls indicates that total quantities of cell wall RG-II are diminished in both rosette leaves and roots of the *tmn1* mutants compared with Col-0, irrespective of B conditions.

To examine RG-II distribution, RG-II was visualized in cross-sections of PRs grown on solid media by labelling with an RG-II antibody. In meristematic zone of roots, signal intensities in the stele and the cortex periphery were decreased in the *tmn1* mutants compared with Col-0, whereas there were no apparent differences in the epidermis (Fig. 5C). In elongation zone of the roots, the signals in the cross-sections were entirely reduced in the *tmn1* mutants compared to Col-0 (Fig. 5D). This observation supports that RG-II amounts were reduced in both the meristematic and elongation zones of the roots in the *tmn1* mutants.

These results demonstrate that AtTMN1 is necessary for maintenance of the quantity of RG-II in cell walls. Because these reductions are consistent with the reductions of dry weight under all B conditions, it is highly likely that reduced quantities of RG-II, which cause a reduction in borate-dimerized RG-II, are at least partly responsible for the biomass reductions and altered growth responses observed in the *tmn1* mutants under severely low B conditions.

### Quantities of cell wall RG-I were also reduced in the rosette leaves of *tmn1* mutants

To investigate whether cell wall components other than RG-II were affected by the lack of AtTMN1, the quantities of 10 monosaccharides were determined in leaf cell walls of plants grown with 100 μM B under short days. Regardless of the presence of amylase treatment, rhamnose content was significantly reduced by 21.2–27.3% in the *tmn1* mutants, compared with Col-0 (*P*<0.001; Table 2, Supplementary Table S2). The quantity of GalA (a substantial component of HG) was significantly reduced by 6.5% in *tmn1-1* cell walls treated with amylase, and by 14.8% and 17.3% in *tmn1-1* and *tmn1-2* cell walls without amylase treatment, respectively (*P*<0.05 ; Table 2, Supplementary Table S2). Glucose, xylose, mannose and glucuronic acid contents were significantly increased in the *tmn1* mutants, compared with Col-0, in cell walls treated with amylase (*P*<0.05; Table 2, Supplementary Table S2). Because rhamnose in cell walls is largely derived from RG-I, these reductions in rhamnose contents suggested that quantities of RG-I were also reduced in the cell walls of the *tmn1* mutants. Additionally, quantities of HG may have decreased slightly in the *tmn1* mutants. This observation suggests that AtTMN1 functions in the deposition of pectic polysaccharides in cell walls, especially RG-II and RG-I.

**Table 2.** Reduced rhamnose contents in *tmn1* mutant cell walls treated with amylase.

Plant line	Mol% of monosaccharides in rosette leaf cell wall									
	Rha	GalA	Glc	Xyl	Man	Ara	Gal	Fuc	mGlcA	GlcA
Col-0	7.75±0.11	41.6±0.67	24.2±0.60	5.84±0.07	1.91±0.05	9.41±0.23	7.63±0.08	1.23±0.02	0.27±0.02	0.14±0.01
*tmn1-1*	5.65±0.14^***^	38.9±1.12^*^	27.9±0.74^***^	6.26±0.17^**^	2.16±0.12^**^	9.73±0.22	7.48±0.21	1.41±0.05^***^	0.33±0.06	0.20±0.02^***^
*tmn1-2*	5.83±0.13^***^	40.1±1.75	27.7±0.59^***^	6.22±0.15^**^	2.13±0.10^*^	8.99±0.54	7.16±0.37	1.33±0.08^*^	0.32±0.04	0.18±0.01^*^
*tmn1-3*	5.80±0.14^***^	40.9±1.65	27.4±0.57^***^	6.27±0.08^**^	2.09±0.07^*^	8.77±0.61	7.04±0.27^*^	1.30±0.04	0.32±0.18	0.17±0.01^*^

The 10 monosaccharides contents were determined in rosette leaf cell walls treated with amylase. The molar percentages were calculated by dividing with total detected monosaccharide contents. Plants were grown hydroponically under 100 μM B for 44 d under short days. Rosette leaves of six plants were harvested and homogenized to create one independent cell wall sample. After amylase treatment, the quantities of 10 monosaccharides were determined. Data are means ± SD from four independent rosette leaf cell walls treated with amylase in Col-0, *tmn1-1*, *tmn1-2*, and *tmn1-3*. Asterisks indicate significant differences between Col-0 and the *tmn1* mutants (*P<0.05, **P<0.01, ***P<0.001; Dunnett’s multiple comparison test). Rha, l-rhamnose; GalA, d-galacturonic acid; Glc, d-glucose; Xyl, d-xylose; Man, d-mannose; Ara, l-arabinose; Gal, d-galactose; Fuc, l-fucose; m-GlcA, 4-*O*-methyl-d-glucuronic acid; GlcA, d-glucuronic acid.

### GFP-AtTMN1 BFA responses were largely similar to those of Golgi proteins

**Figure 6.**
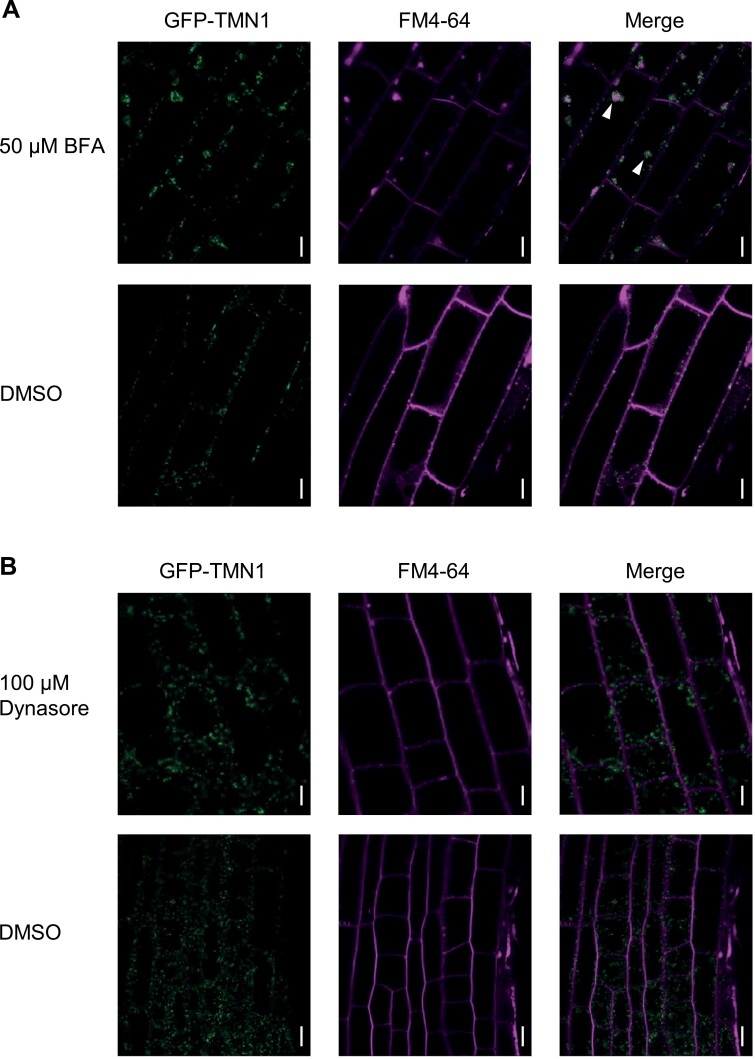
Golgi localization of GFP-AtTMN1 in the presence of BFA and Dynasore. (A) Sub-cellular localization of GFP-AtTMN1 under BFA treatment. Five-d-old transgenic plants carrying *proAtTMN1:SP(AtTMN1)-GFP-AtTMN1genome* (#1) under 100 μM B were incubated with 2 μM FM4-64 for 5 min and then with 50 μM cycloheximide for 30 min, followed by 50 μM BFA and cycloheximide for 60 min. Arrowheads indicate ring-like signals of GFP-AtTMN1. (B) Sub-cellular localization of GFP-AtTMN1 under Dynasore treatment. Five-d-old *proAtTMN1:SP(AtTMN1)-GFP-AtTMN1genome* transgenic plants (#1) under 100 μM B conditions were treated with 100 μM Dynasore for 90 min and then with 1 μM FM4-64 for 30 s. Scale bars =10 μm. Similar patterns were observed in the four independent transgenic lines.

It has been reported that AtTMN1 is localized in the Golgi apparatus in Arabidopsis (Gao *et al.*, 2012). RG-I and RG-II structures are synthesized at the Golgi apparatus by a variety of glycosyltransferases (Driouich *et al.*, 2012); packaged pectic polysaccharides are then delivered into the apoplast matrix via two *trans*-Golgi network derived pathways (Sinclair *et al.*, 2018). Therefore, we hypothesized that AtTMN1 functions in pectin biosynthesis in the Golgi apparatus and/or in secretion to the apoplast. To gain insight into the molecular function of AtTMN1, GFP-AtTMN1 localization was observed in the presence of exocytosis and endocytosis inhibitors. Under treatment with 50 μM BFA (an exocytosis inhibitor) accompanied by cycloheximide for 60 min, ring-like localization of GFP-AtTMN1 was mainly observed on the BFA compartment (Robinson *et al.*, 2008) of FM4-64 (an endocytosis tracer; Fig. 6A). The distribution pattern of GFP-AtTMN1 was similar to the pattern of ST-mRFP (Naramoto *et al.*, 2014), confirming the localization of GFP-AtTMN1 mainly in the Golgi apparatus. Furthermore, under 90 min treatment with 100 μM Dynasore (a dynamin-dependent endocytosis inhibitor), GFP-AtTMN1 fluorescence signals did not show co-localization with FM4-64 on the plasma membrane (Fig. 6B). This suggested the likelihood that most of the GFP-AtTMN1 was neither localized to trafficking systems nor targeted to the plasma membrane. From these observations of GFP-AtTMN1 localization, we inferred that AtTMN1 plays a role in pectin biosynthesis in the Golgi apparatus, rather than in trafficking pectin to the apoplast.

Furthermore, genes co-expressed with *AtTMN1* (*r*>0.6; 488 genes; Supplementary Table S3) include at least 20 published genes proven to be related to pectin biosynthesis and modification (Supplementary Table S3). Enrichment analysis revealed that terms related to the Golgi apparatus, pectin, and primary cell wall were over-represented among the co-expressed genes (Supplementary Table S4). These data support our conclusion that AtTMN1 is involved in the pectin deposition pathway.

## Discussion

### AtTMN1 plays a role in pectic polysaccharide deposition in cell walls

In this study, AtTMN1 was identified as a novel component required for pectin deposition, which governs cell elongation and consequently overall plant growth. We screened Arabidopsis mutants with impaired growth responses to low B nutrition, based on prior observations that deficits in RG-II synthesis lead to altered responses to B nutrition (O’Neill *et al.*, 2001; Sechet *et al.*, 2018). In the *tmn1* mutants, quantities of RG-II, represented by 2-keto-3-deoxy sugar concentrations, and RG-I, represented by rhamnose contents, were decreased by 20–30% in leaf and root cell walls under all B treatments (Fig. 5; Table 2), which demonstrated that AtTMN1 was essential for normal pectin deposition in cell walls.

Considering the importance of RG-II and RG-I in cell growth (O’Neill *et al.*, 2001; Oomen *et al.*, 2002; Delmas *et al.*, 2008; Kobayashi *et al.*, 2011; Liu *et al.*, 2011), these results suggested that reductions of RG-II and RG-I contents were the main causes of cell elongation inhibition (Fig. 2C, D). Meanwhile, the *tmn1* mutants exhibited no apparent abnormalities of cell division (Fig. 2E-H) despite the reduced RG-II amounts in the meristematic zone of the PRs (Fig. 5C), implying that the RG-II reduction in the *tmn1* mutants was not so severe as to cause defects in cell division. This view is supported by the observation that reduced B-crosslinked RG-II under B deficiency primarily impairs cell elongation (Camacho-Cristóbal *et al.*, 2015). Thus, it is assumed that the cell elongation inhibition rather than the defect of cell division resulted in whole-plant growth and developmental impairment in the *tmn1* mutants. This is demonstrated by a reduction of dry weight in the vegetative growth stages under all tested B conditions, as well as impaired reproductive growth under sufficient B supply (100 µM and 500 μM; Fig. 3; Supplementary Fig. S4). The assumption is supported by the prior observation that a 20% reduction in borate dimerized RG-II likely caused dwarfism in Arabidopsis *mur1* and *GDP-**d**-mannose 3,5-epimerase*-silenced tomato plants with impaired RG-II structure (O’Neill *et al.*, 2001; Voxeur *et al.*, 2011).

### Altered pectin affects growth under limited B conditions

Under severely low B, the *tmn1* mutants displayed alleviation of root elongation inhibition and reproductive growth failure compared with WT plants (which exhibited reduced growth) (Figs 1, 3Supplementary Fig. S4). In roots, this phenomenon was caused by increment of cell elongation in the *tmn1* mutants (Fig. 2A, B), in contrast to inhibition of cell elongation under B sufficiency (Fig. 2C, D). Cell elongation is generally inhibited under low B conditions, and pollen tube elongation is suppressed by B depletion, resulting in plant sterility (Dell and Huang, 1997; Huang *et al.*, 2000; Wang *et al.*, 2003). Therefore, we conclude that the alleviation of cell elongation inhibition in the corresponding tissues explained the *tmn1* mutant phenotypes under severely low B conditions.

Cell enlargement in specific tissues of the *tmn1* mutants, compared with Col-0, may have been caused by reduction of overall pectin content, through cell wall loosening under severely limited B, when cell elongation was severely inhibited in Col-0. In roots, reduction of Ca^2+^ cross-linked HG (Supplementary Fig. S6C) could cause cell wall loosening and result in increased cell elongation in the mutants under severely low B. However, based on the assumption that RG-II cross-linking by borate is a primary determinant for growth under low B, reduced RG-II content might be a major contributing factor in the increment of cell elongation in the *tmn1* mutants under limited B supply. In mutant cell walls under severely low B, B concentrations and total RG-II content were reduced, compared with WT plants (Figs 4, 5); this suggested that absolute quantities of dRG-II-B and monomeric RG-II had decreased. Considering that B deficiency inhibits cell elongation in WT through the reduction of dRG-II-B and increase in monomeric RG-II contents compared with plants under sufficient B supply, the reduction in monomeric RG-II, but not in dRG-II-B, in the *tmn1* mutants likely resulted in the alleviation of cell elongation inhibition under severely low B conditions. This hypothesis is consistent with the findings in a previous study, where differences in tolerance to low B among rapeseed genotypes were reportedly defined by low quantities of pectin, presumably in the form of monomer RG-II (Zhou *et al.*, 2017). Our results further suggest that altered pectin, mainly RG-II, modifies plant growth under limited B supply.

Several homologs of the *Catharanthus roseus* receptor-like kinase subfamily have been shown to play regulatory roles in cell expansion by binding cell wall polysaccharides or glycosylated proteins (Nissen *et al.*, 2016). These proteins (e.g., FER, a receptor kinase that interacts with the pectin polysaccharide backbone) may sense the disruption of pectin cross-linking by B and Ca^2+^ (Feng *et al.*, 2018); these proteins may perceive monomeric RG-II and modulate cell elongation under limited B conditions.

### The TMN protein family shares cell adhesion functions among eukaryotes

TMN proteins are conserved as the transmembrane 9 superfamily (TM9SF) among eukaryotes; some of these proteins have been characterized in organisms other than plants. Down-regulation of human TM9SF4, which belongs to the other cluster from AtTMN1 in the phylogenetic tree separated into two clusters (Hegelund *et al.*, 2010), has been shown to reduce adhesion of myelomonocytic cells to fibronectin (Paolillo *et al.*, 2015). In *Drosophila,* a null mutant of putative phagocytic receptor 1a (*Phg1a*; an ortholog of *TM9SF4*) showed defective phagocytosis against wasp eggs (Bergeret *et al.*, 2008). Given that haemolymph cells can recognize and attach to invaders, the *Drosophila phg1a* mutant may lose the capacity for cellular attachment under these conditions.

In *Dictyostelium*, a unicellular organism, the number of cells adhering to hydrophilic glass surfaces was reduced in a *Phg1A*-knockout mutant (Cornillon *et al.*, 2000). Similarly, the triple mutant *S. cerevisiae tmn1 tmn2 tmn3* displayed reduced adhesion to solid yeast extract–peptone–dextrose medium surfaces and a concurrent filamentous growth defect (Froquet *et al.*, 2008), such that single yeast cells assembled into a multicellular form in response to nitrogen starvation (Mösch and Fink, 1997; Cullen and Sprague, 2012). Together, these data show that *TMN* family members are essential for intercellular attachment among eukaryotes, including both multicellular and unicellular organisms, although the regulation systems and molecules involved in cell attachment are distinct among organisms.

The *AtTMN1* mutations impaired the detachment of the proximal end of root cap, consistent with its expression (Supplementary Figs S1, S2). The previous studies on loss-of-function mutants in pectin biosynthesis and degradation demonstrated that proper pectin metabolism is required for root cap maturation (Durand *et al.*, 2009; Bennett *et al.*, 2010; Kamiya *et al.*, 2016). Although the *tmn1* phenotype does not agree with a hypothesis that reduced amounts of pectin including HG promotes the root cap detachment, it is suggested that Arabidopsis TMN1 contributes to appropriate intercellular attachment of columella cells through proper pectin deposition. Considering that normal pectin is required for plant cell adhesion (Bouton *et al.*, 2002; Iwai *et al.*, 2002), it is believed that *AtTMN1* in plants also contributes to the biological processes involved in cellular attachment mediated by pectin deposition.

### AtTMN1 might be involved in vesicular trafficking for pectin biosynthesis

Based on analyses of *TMN* knockout mutants and overexpression lines, it is suggested that TMN families control the amounts of cargo molecules at the plasma membrane in *Dictyostelium*, *Drosophila* and human (Froquet *et al.*, 2012; Perrin *et al.*, 2015a, b). Furthermore, molecular interactions between the cargo molecules and TMN proteins suggest that TMN proteins function as cargo receptors in the vesicular trafficking pathway to secrete them into the cell surface. 

In the current study, we found that Arabidopsis TMN1 controls RG-II and RG-I deposition in the apoplast and that a major portion of TMN1 displayed the pattern of Golgi-localized proteins. The cytoplasmic tail of the C-terminus in AtTMN1 possesses the KXD/E motif, a Golgi retention signal, which is conserved among other TMN homologs in eukaryotes (Gao *et al.*, 2014). Furthermore, AtTMN1 interacts with Sec21 (a coat protein complex I subunit) and Sec24 (a coat protein complex II subunit) to mediate retrograde and anterograde transport, respectively, between the Golgi apparatus and endoplasmic reticulum (Gao *et al.*, 2012); thus, AtTMN1 is presumably involved in a vesicular trafficking system. Given the Golgi localization of a series of proteins, including glycosyltransferases and nucleotide sugar transporters required for pectin biosynthesis (Mohnen, 2008; Nikolovski *et al.*, 2012), and the interaction of AtTMN1 with coat protein complex subunits, AtTMN1 may transport these proteins required for RG-II and RG-I synthesis from the endoplasmic reticulum to the Golgi. On the other hand, we do not rule out a possibility that Arabidopsis TMN1 is involved in trafficking of biosynthesized pectin from the Golgi to apoplast, considering that ECHIDNA, YPT/RAB GTPase interacting Protein 4a and b are localized on the *trans*-Golgi network but not plasma membrane; they function in secretion of HG, RG-I and xyloglucan (Gendre *et al.*, 2013; McFarlane *et al.*, 2013). Therefore, it is possible that a minor portion of AtTMN1 localized at the *trans*-Golgi network contributes to pectin transport.

In conclusion, the results of this study demonstrate that the plant TMN family plays roles in RG-II and RG-I pectic polysaccharide deposition for normal cell elongation. We also found that the pectin synthesis mutant exhibited reduced sensitivity to low B supply in terms of cell elongation. Our findings shed light on the mechanisms of growth regulation, which are dependent on changes in pectin content under low B conditions.

## Supplementary data

The following supplementary data are available at *JXB* online.

Fig. S1. Expression of GFP-AtTMN1 in root tips.

Fig. S2. Failed cellular detachment of root caps in the *tmn1* mutants under sufficient B condition.

Fig. S3. Reduction tendency of root tip thickness in *tmn1-1* and *tmn1-2*.

Fig. S4. Restoration of *tmn1* mutant reproductive growth under severely low B condition.

Fig. S5. No substantial reduction in total B concentration in the *tmn1* mutants under sufficient B conditions.

Fig. S6. No consistent patterns of changes in cell wall Ca concentrations of the *tmn1* mutants.

Table S1. F_2_ segregating population derived from F_1_ of mutants nos.19 or 45 crossed with L*er*.

Table S2. Reduced rhamnose and galacturonic acid contents in *tmn1* mutant cell walls without amylase treatment.

Table S3. Genes co-expressed with *AtTMN1*.

Table S4. Enrichment analysis results for genes co-expressed with *AtTMN1*.

Table S5. Primers used in this study.

erab065_suppl_Supplementary-Figure-S1-6_Tables-1-2-5

erab065_suppl_Supplementary-Tables-S3_S4

## Data Availability

All data supporting the findings of this study are available within the paper and within its supplementary data published online.

## Abbreviations

AIR: alcohol-insoluble residue
B: boron
BFA: brefeldin A
DAS: day after sowing
GFP: green fluorescent protein
HG: homogalacturonan
Phg1a: putative phagocytic receptor 1a
PI: propidium iodide
PR: primary root
RG-I: rhamnogalacturonan I
RG-II: rhamnogalacturonan II
TMN1: transmembrane nine 1
TM9SF: transmembrane 9 superfamily
