# Supplementary material for: Golgi-localized membrane protein AtTMN1/EMP12 functions in the deposition of rhamnogalacturonan II and I for cell growth in Arabidopsis
Source: J Exp Bot. 2021 Feb 15;72(10):3611–29. doi: 10.1093/jxb/erab065 (PMC8096605; doi:10.1093/jxb/erab065)
Supplement: erab065_suppl_Supplementary-Figure-S1-6_Tables-1-2-5 [file erab065_suppl_supplementary-figure-s1-6_tables-1-2-5.pdf]

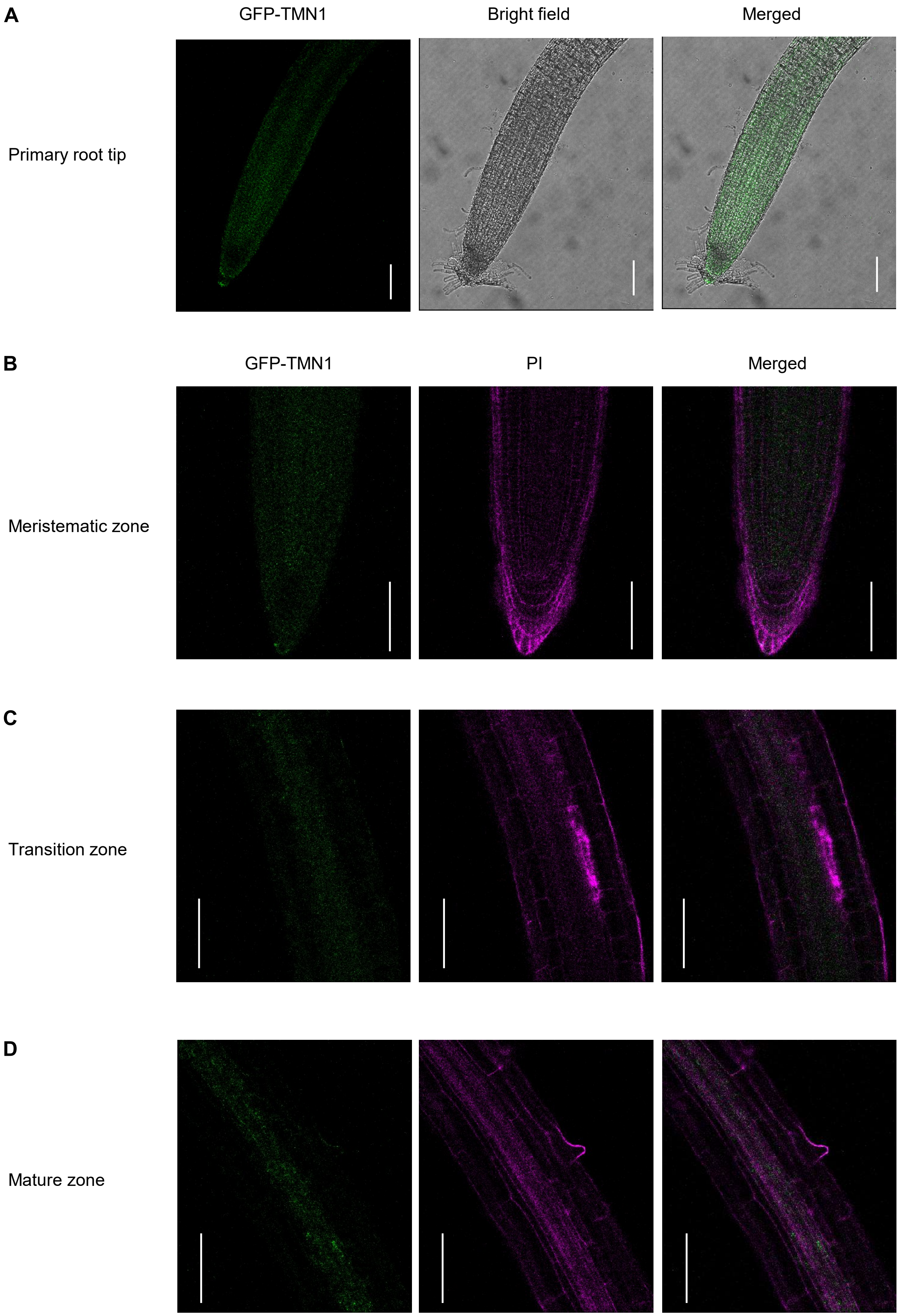

Supplementary Figure S1. Expression of GFP-AtTMN1 in root tips.
Representative images of GFP-AtTMN1 fluorescence of root tip (A), meristematic zone (B), transition zone (C) and mature zone in PRs. Five-d-old transgenic plants carrying *proAtTMN1:SP(AtTMN1)-GFP-AtTMN1genome* (#1) under 100 μM B condition. Scale bars =100 μm. Their fluorescence in (B), (C) and (D) was enhanced using Leica Application Suite Advanced Fluorescence Lite.

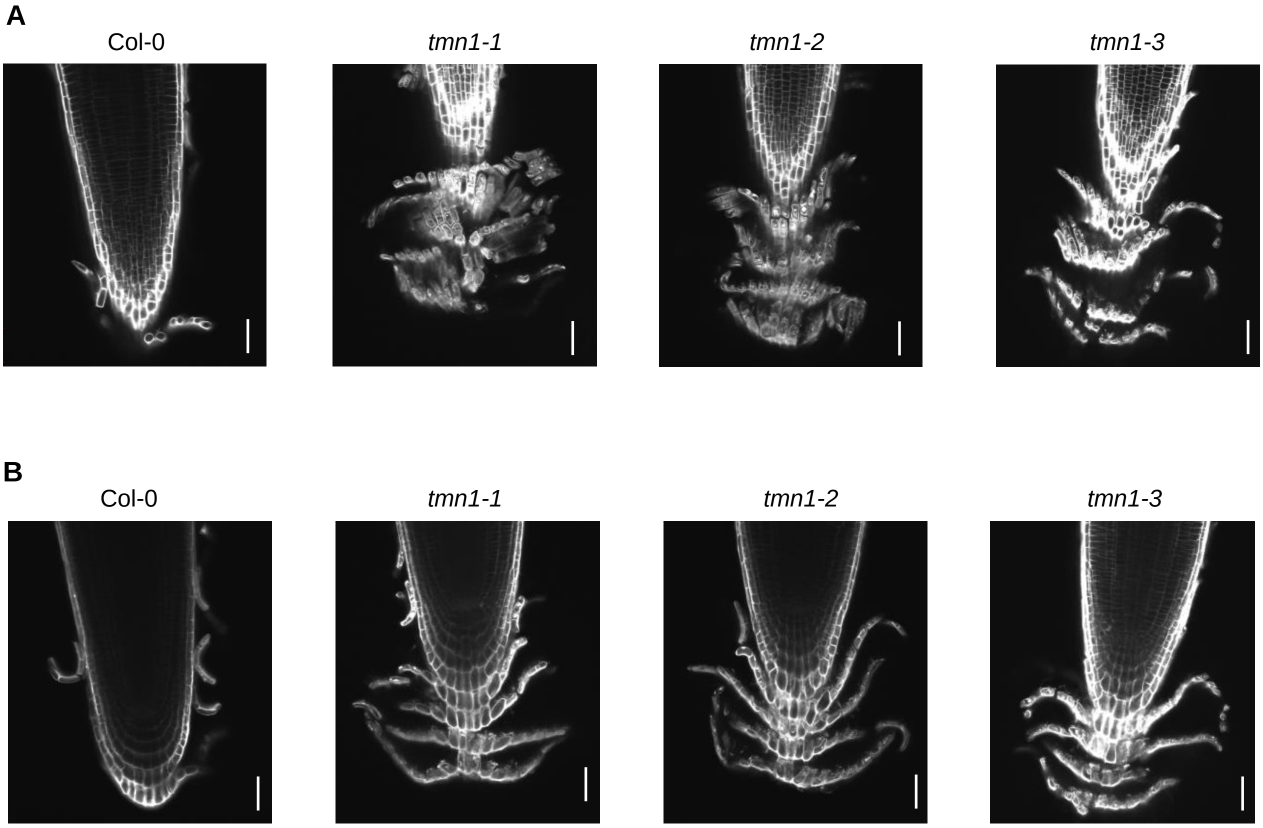

Supplementary Figure S2. Failed cellular detachment of root caps in the *tmn1* mutants under sufficient B condition.
Representative confocal images taken at positions of root surface (A) and focusing on centre (B) in 14-d-old seedlings of Col-0, *tmn1-1*, *tmn1-2* and *tmn1-3* under 100 μM B condition. Roots stained with PI. Scale bars =50 μm.

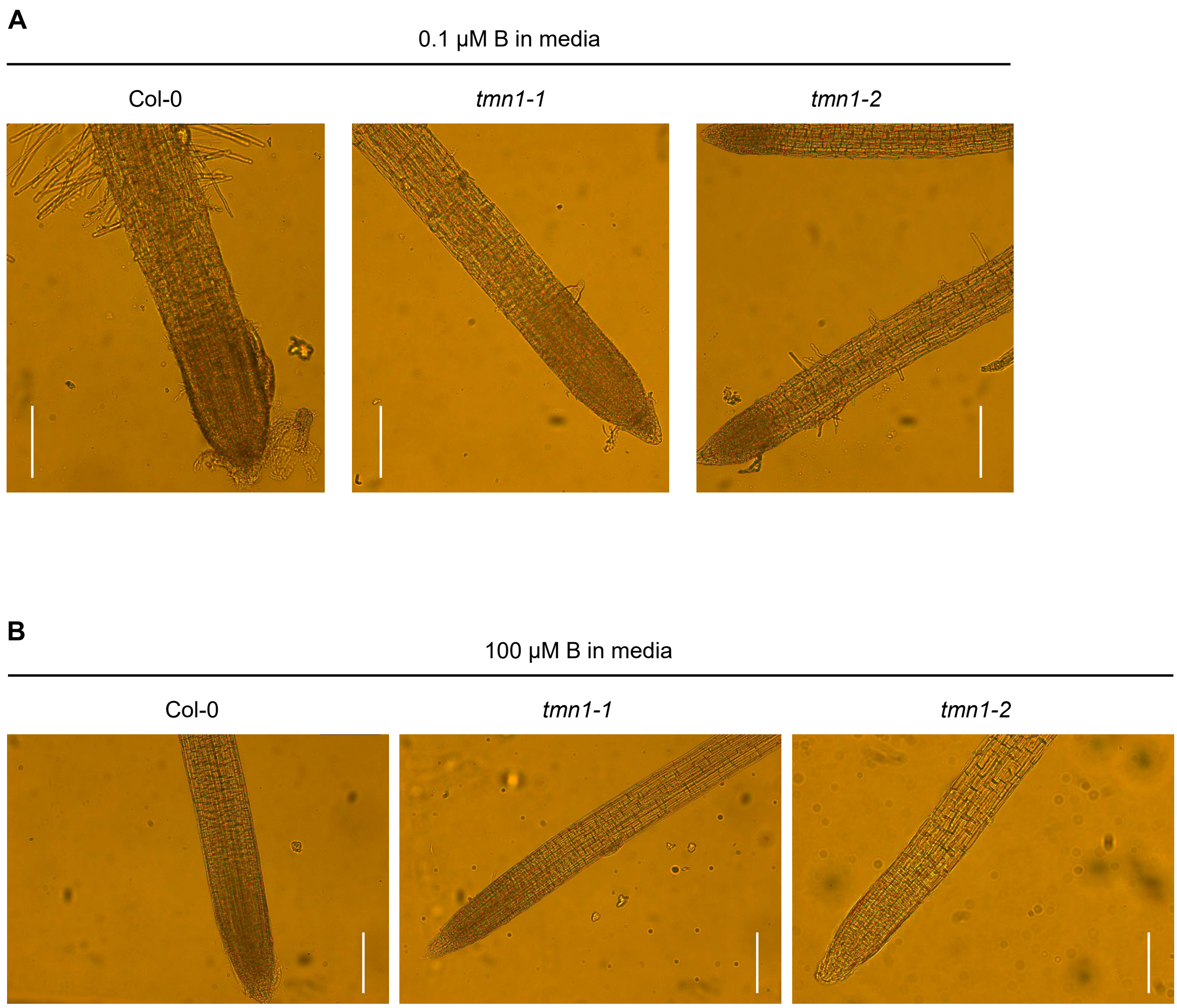

Supplementary Figure S3. Reduction tendency of root tip thickness in *tmn1-1* and *tmn1-2.*
Root tip thickness of Col-0, *tmn1-1* and *tmn1-2*. The plants were grown hydroponically for 44 d under short-day conditions. The longest roots of each bundle were observed under 0.1 (A) and 100 µM B conditions. Bright field images were shown. Scale bars =100 µm.

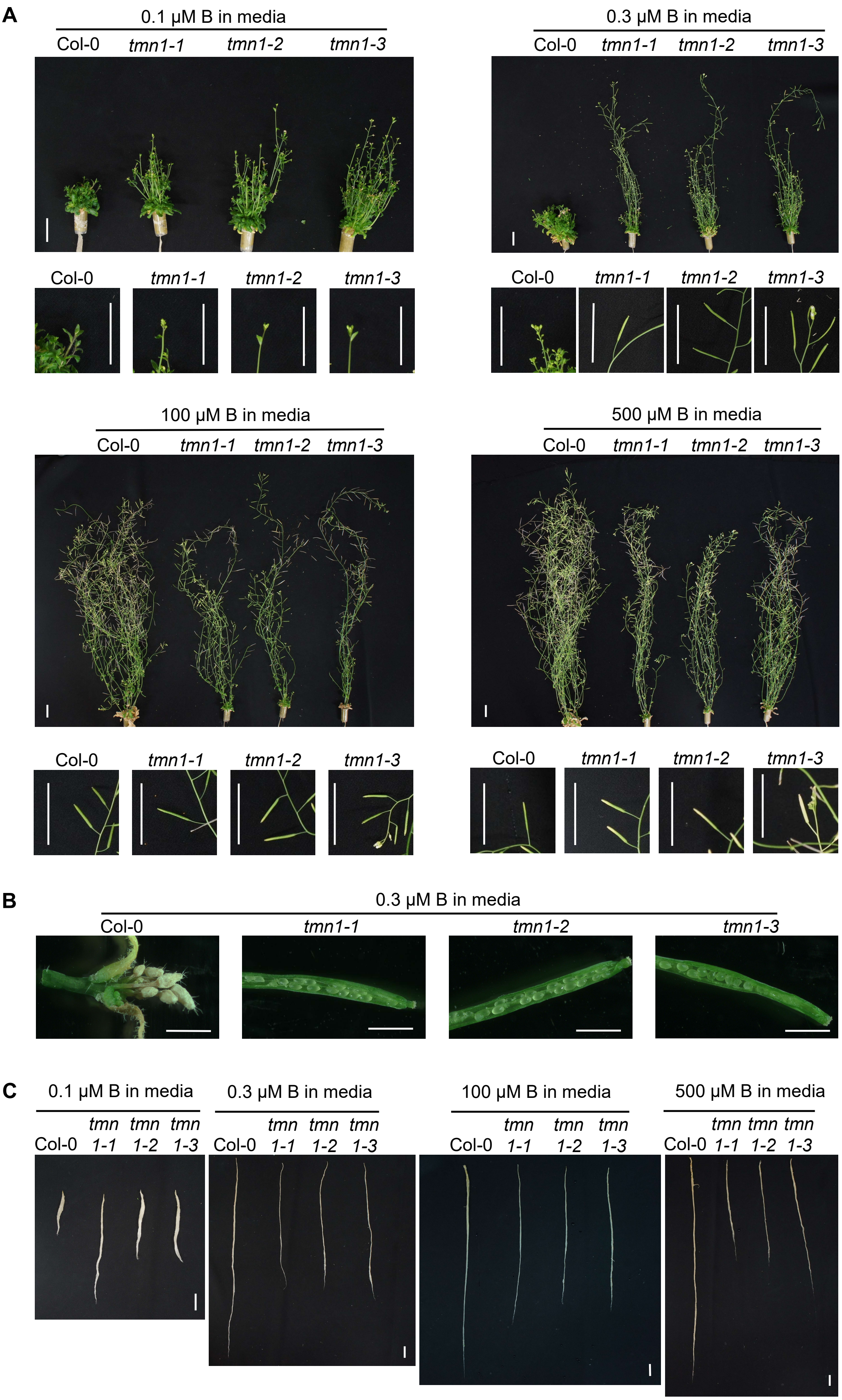

Supplementary Figure S4. Restoration of *tmn1* mutant reproductive growth under severely low B condition. (A) Aerial portions of Col-0, *tmn1-1*, *tmn1-2* and *tmn1-3* grown hydroponically for 75 d under 0.1, 0.3, 100 and 500 μM B conditions under long-day conditions. Scale bars =20 mm. (B) Magnified pictures of a floral organ and siliques in Col-0, *tmn1-1*, *tmn1-2* and *tmn1-3* grown under 0.3 μM B condition. Scale bars =10 mm. (C) Roots of 75-d-old plants in Col-0, *tmn1-1*, *tmn1-2* and *tmn1-3* under 0.1, 0.3, 100 and 500 μM B conditions. Scale bars =20 mm.

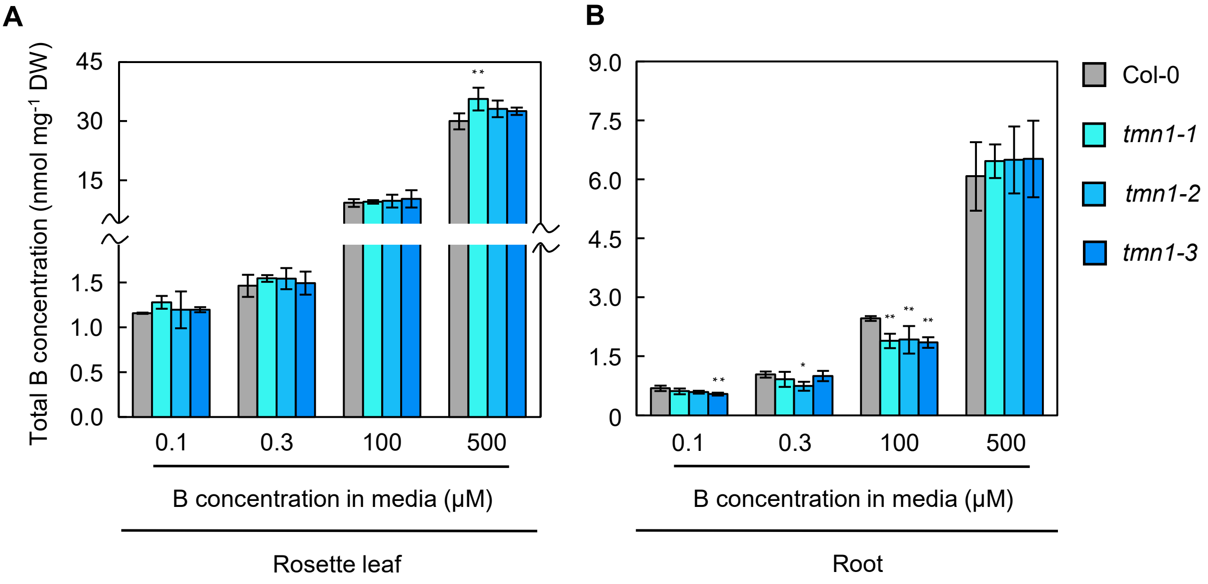

Supplementary Figure S5. No substantial reduction in total B concentration in the *tmn1* mutants under sufficient B conditions.

Total B concentrations in rosette leaves (A) and roots (B) of Col-0, *tmn1-1*, *tmn1-2* and *tmn1-3*. The plants were grown hydroponically under 0.1, 0.3, 100 and 500 μM B conditions for 45 d under short-day conditions. Values represent means ± SD from four independent samples for rosette leaves and roots. Significant differences between Col-0 and the *tmn1* mutants under each B condition are indicated as **P*<0.05, ***P*<0.01 (Dunnett's multiple comparison test).

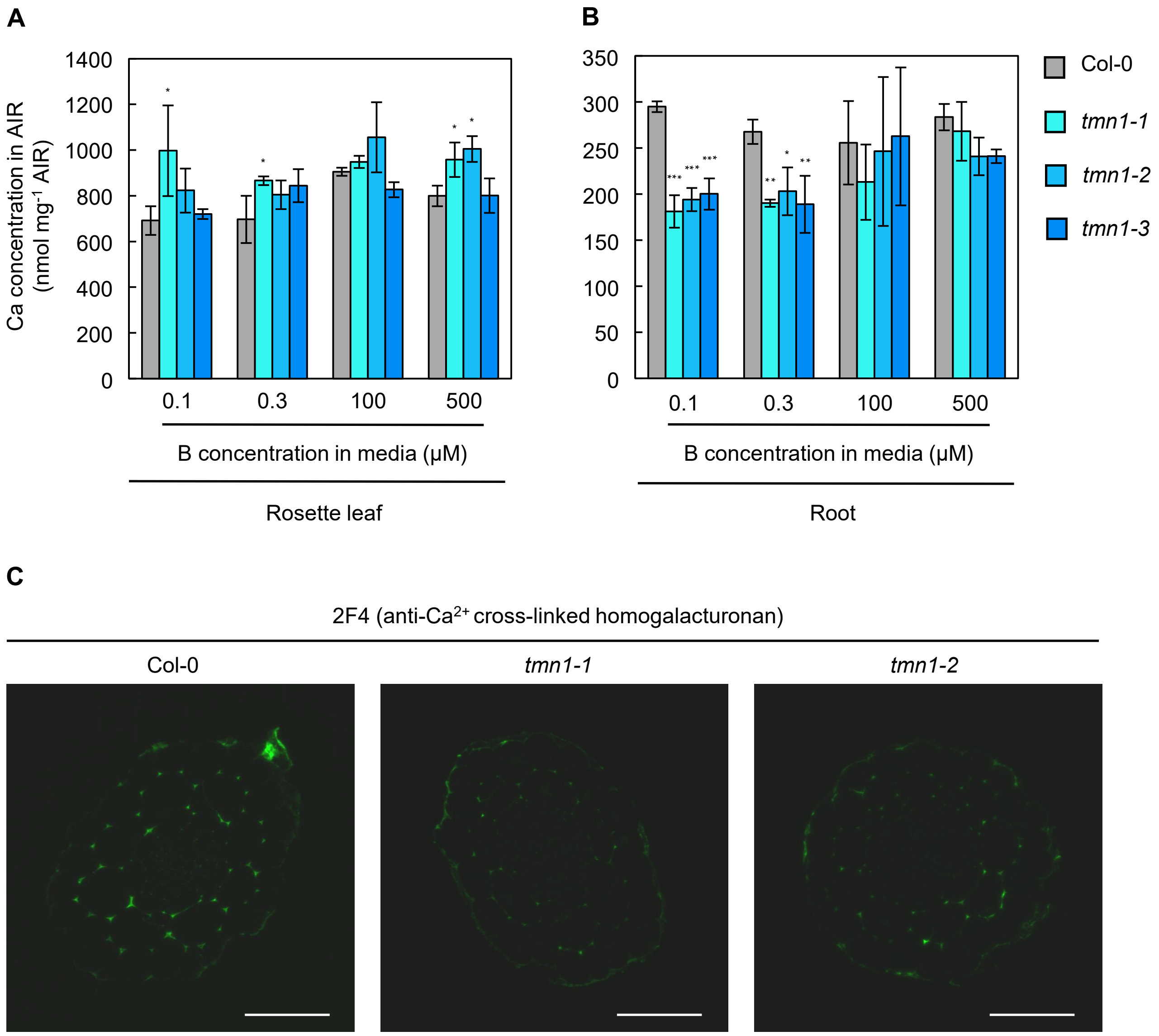

Supplementary Figure S6. No consistent patterns of changes in cell wall Ca concentrations of the *tmn1* mutants.

Ca concentrations in rosette leaf (A) and root (B) cell walls of Col-0, *tmn1-1*, *tmn1-2* and *tmn1-3*. The plants were grown hydroponically under 0.1, 0.3, 100 and 500 μM B conditions for 44 d under short-day conditions. For each cell wall sample, six individual plants for rosette leaves and four-nine plants for roots were harvested and homogenized. Values represent means ± SD from three and four independent cell wall samples for rosette leaves and roots, respectively. Significant differences between Col-0 and the *tmn1* mutants under each B condition are indicated as *P<0.05, **P<0.01, ***P<0.001 (Dunnett's multiple comparison test). AIR, alcohol-insoluble residue. (C) Immunohistochemistry of root cross-sections in elongation zone of PRs with an anti-Ca^{2+} cross-linked homogalacturonan antibody (2F4). Plants were grown on solid media under 0.1 μM B condition for 5 d. Scale bars =50 μm.

Supplementary Table S1. F2 segregating population derived from F1 of mutants nos.19 or 45 crossed with L*er*.

Plants were grown on solid media under 0.03 μM B condition for 11-13 d under long-day conditions. The number of plants showing increased PR elongation compared to Col-0 or similar PR lengths to Col-0 was counted.

F1 genotype	The number of plants with mutant phenotypes	The numbers of plants similar to Col-0	Total numbers of germinated F2 population
No.19/ L*er*	26	106	132
No.45/ L*er*	51	294	345

Supplementary Table S2. Reduced rhamnose and galacturonic acid contents in *tmn1* mutant cell walls without amylase treatment.

The 10 monosaccharides contents were determined in rosette leaf cell walls treated without amylase. The molar percentages were calculated by dividing with total detected monosaccharide contents. Plants were grown hydroponically under 100 µM B condition for 44 d under short-day conditions. Rosette leaves of six plants were harvested and homogenized to create one independent cell wall sample. The quantities of 10 monosaccharide were determined. Data are means ± SD from four independent rosette leaf cell walls in Col-0, *tmn1-1*, *tmn1-2* and *tmn1-3*. Asterisks indicate significant differences between Col-0 and the *tmn1* mutants (**P*<0.05, ***P*<0.01, ****P*<0.001; Dunnett's multiple comparison test). Rha, L-rhamnose; GalA, D-galacturonic acid; Glc, D-glucose; Xyl, D-xylose; Man, D-mannose; Ara, L-arabinose; Gal, D-galactose; Fuc, L-fucose; m-GlcA, 4-*O*-methyl-D-glucuronic acid; GlcA, D-glucuronic acid.

	Mol% of monosaccharides in rosette leaf cell wall									
Plant line	Rha	GalA	Glc	Xyl	Man	Ara	Gal	Fuc	mGlcA	GlcA
Col-0	6.27 ± 0.18	31.4 ± 1.18	38.6 ± 1.61	3.70 ± 0.10	2.03 ± 0.10	8.80 ± 0.25	7.67 ± 0.09	1.15 ± 0.04	0.17 ± 0.01	0.17 ± 0.02
tmn1-1	4.56 ± 0.18***	26.8 ± 2.08**	43.0 ± 2.01	3.91 ± 0.16	2.36 ± 0.13	9.71 ± 0.44	7.86 ± 0.25	1.41 ± 0.05***	0.29 ± 0.03**	0.17 ± 0.12
tmn1-2	4.94 ± 0.34***	26.0 ± 2.53**	45.8 ± 3.44**	3.36 ± 0.57	2.75 ± 0.30***	8.57 ± 0.36	6.92 ± 0.65*	1.30 ± 0.08**	0.27 ± 0.06**	0.07 ± 0.10
tmn1-3	4.57 ± 0.15***	29.4 ± 0.58	41.7 ± 1.81	4.06 ± 0.17	2.25 ± 0.08	8.74 ± 0.74	7.53 ± 0.32	1.27 ± 0.04*	0.19 ± 0.04	0.27 ± 0.09

Supplementary Table S5. Primers used in this study

Primer No.	Name	Sequence (5’ > 3’)	Purpose
P1	*tmn1-1*_dCAPS_For	GCTGGTTTATTCAGGTAAACATCG	Genotyping of *tmn1-1*
P2	*tmn1-1*_dCAPS_Rev	AGGGAGACGACTGATGGAGT	
P3	*tmn1-2*_CAPS_For	ACCTTTGTGGGGTATGAACTT	Genotyping of *tmn1-2*
P4	*tmn1-2*_CAPS_Rev	TGCTTAATGGCGTGGTAACAA	
P5	WiscDsLoxHS_LB	TGATCCATGTAGATTTCCCGGACATGAAG	Genotyping of *tmn1-3*
P6	*TMN1*_genome_For	GCACAGGAACGGGATGTAAGT	
P7	*TMN1*_genome_Rev	AGGGAGACGACTGATGGAGT	
P8	*TMN1*_Coding sequence1_For	ATGCCGTCTTCCTCCTCCGCCG	Detection of full-length *TMN1* transcript
P9	*TMN1*_Coding sequence1_Rev	GTCGCACTTGATGTTTCTGTAG	
P10	*Actin1*_5’ UTR_For	CTCCGATTTGATGGAGTCTGGT	Detection of full-length *Actin1* transcript
P11	*Actin1*_3’ UTR_Rev	TGAAGAGGAAGAGGTGTGTACT	
P12	*TMN1*_Promoter1_For	CACCTTCAGACGTGGAGCAGGACT	Cloning of full-length *TMN1* genome
P13	*TMN1*_Terminater_Rev	CGATGTGTGGGCTCTTGTGT	
P14	*TMN1*_Promoter2_For	GCCTAAACAAACTCCCATCATG	Cloning of TMN1 signal peptide
P15	*TMN1*_N-terminus_Rev	ACCATAGAACCACCACCACCATCGGATGCGAACGTAGGAG	
P16	*GFP*_Coding sequence_For	CCGATGGTGGTGGTGGTTCTATGGTGAGCAAGGGCGAGGA	Cloning of *GFP* coding sequence
P17	*GFP*_Coding sequence_Rev	AGATCCTCCTCCTGATCCTCCTCCCTTGTACAGCTCGTCCATGC	
P18	*TMN1*_Coding sequence2_For	GGAGGAGGATCAGGAGGAGGATCTTCAGATCACAAGGTAAGACT	Cloning of *TMN1* coding sequence
P19	*TMN1*_Coding sequence2_Rev	CGAGAATCTCAAACCAACATC	
